# Association of Soluble HLA-G Plasma Level and *HLA-G* Genetic Polymorphism With Pregnancy Outcome of Patients Undergoing *in vitro* Fertilization Embryo Transfer

**DOI:** 10.3389/fimmu.2019.02982

**Published:** 2020-01-14

**Authors:** Izabela Nowak, Karolina Wilczyńska, Paweł Radwan, Andrzej Wiśniewski, Rafał Krasiński, Michał Radwan, Jacek R. Wilczyński, Andrzej Malinowski, Piotr Kuśnierczyk

**Affiliations:** ^1^Laboratory of Immunogenetics and Tissue Immunology, Department of Clinical Immunology, Ludwik Hirszfeld Institute of Immunology and Experimental Therapy, Polish Academy of Sciences, Wrocław, Poland; ^2^Department of Reproductive Medicine, Gameta Hospital, Rzgów, Poland; ^3^Biogeno—Regional Science-Technology Centre, Podzamcze, Poland; ^4^Faculty of Health Sciences, The State University of Applied Sciences in Płock, Płock, Poland; ^5^Department of Surgical and Oncological Gynecology, Medical University of Łódź, Łódź, Poland; ^6^Department of Surgical, Endoscopic and Oncologic Gynecology, Polish Mothers' Memorial Hospital–Research Institute, Łódź, Poland

**Keywords:** HLA-G polymorphism, sHLA-G, *in vitro* fertilization embryo transfer, recurrent implantation failure, fresh or frozen cycle, ovarian stimulation protocol

## Abstract

Infertility is currently a growing problem observed around the world and is estimated to affect between 8 and 12% of reproductive-aged couples worldwide. Artificial reproductive techniques are the last chance for couples seeking their own child. Human leukocyte antigen (HLA)-G expression has been suggested as an immunomodulatory molecule that influences pregnancy outcome. The *HLA-G* gene encodes either membrane-bound or/and soluble proteins. The aim of this study was the evaluation of the role of soluble HLA-G (sHLA-G) and its gene polymorphism in successful implantation after *in vitro* fertilization embryo transfers (IVF-ETs) in different clinical protocols. We tested the *HLA-G* polymorphism in three positions: rs1632947: c.-964G>A; rs1233334: c.-725G>C/T in promoter region; rs371194629: c.^*^65_^*^66insATTTGTTCATGCCT in 3′ untranslated region of exon 8, in 389 patients who underwent IVF-ETs and 320 women with healthy children born after natural conception. Among the patient group, 239 women were with recurrent implantation failure and 117 women had an ongoing pregnancy or a child born after IVF-ET. We found that certain rs1632947-rs1233334-rs371194629 HLA-G haplotypes and diplotypes were associated with infertility, while others were protective. The lowest secretors of sHLA-G were G-C-ins haplotype carriers (37.21 IU/ml), while the highest -G-C-del carriers (73.80 IU/ml). Other haplotype carriers were intermediate secretors. In our study, regardless of possessed haplotype by the patient, 59.73 IU/ml sHLA-G was the threshold value with the best sensitivity (58.82%) and specificity (66.10%) to discriminate patients who achieved and maintained pregnancy from those who did not conceive or they had miscarriage (*p* = 0.0085; likelihood ratio, 1.74; 95% CI = 0.55–0.78). However, we do not exclude that factors other than sHLA-G may also contribute to complications in pregnancy. In addition, we found that IVF patients in cycles when frozen/thawed embryo was transferred secreted higher soluble HLA-G levels than patients with fresh embryo transferred (*p* = 0.021). Moreover, correlation analysis of sHLA-G concentration measured before and after embryo transfer for particular patients indicated short ovarian stimulation with gonadotropin-releasing hormone antagonist as more beneficial than long protocol with gonadotropin-releasing hormone agonist. Our study confirms a role of *HLA-G* polymorphism in infertility and soluble HLA-G in the early stages of pregnancy.

## Introduction

Infertility is currently a growing problem observed around the world and is estimated to affect between 8 and 12% of reproductive-aged couples worldwide (1). Artificial reproductive techniques (ARTs) are the last chance for couples seeking their own child. Despite advances in ART, recurrent implantation failure (RIF) still occurs and affects ~10% of women who have undergone several *in vitro* fertilization embryo transfers (IVF-ETs) (2, 3). The definition of RIF is still not well-defined (2–6) and is described as a lack of pregnancy after at least two consecutive cycles (4), or three embryo transfers (2, 7, 8), or four and more good-quality embryos in a minimum of three fresh or frozen cycles in a woman under the age of 40 years (9), or more than 12 embryos (6). Therefore, a non-invasive biomarker is needed that will indicate as soon as possible whether the embryo transfer is successful or not, or whether there are complications resulting in a miscarriage.

Human leukocyte antigen (HLA)-G expression is mainly restricted to trophoblast cells, and it has been suggested as an immunomodulatory molecule, which has an impact on interactions of different immune cells [decidual natural killer (dNK), T, macrophages] and regulation of cell migration during placental development influencing pregnancy outcome. It means that HLA-G expression is not strictly associated with protection of embryo/fetus against attack of maternal cells, but it is engaged with tissue remodeling. Expressed or secreted HLA-G molecules by extravillous trophoblast cells (EVT) regulate their decidual and endovascular invasion (10–12). Namely, EVT cells progressively replace endothelial cells on the walls of uterine spiral arteries, increasing their diameter that ensures proper blood flow to the intervillous space for fetal nutrition. This process requires the presence of dNK cells, the most numerous cell population at the maternal–fetal interface. Moreover, during interactions with EVT, dNK cells can acquire HLA-G by trogocytosis. Signaling from dNK endosomes stimulates a tolerogenic NK cells activity while maintaining the capacity for antiviral immunity at the maternal–fetal interface (13). HLA-G can interact by its extracellular domains with leukocyte receptors, including CD8, LILRB1, and LILRB2 and the killer cell immunoglobulin-like receptor KIR2DL4 (14).

The *HLA-G* gene encodes either membrane-bound and/or soluble proteins due to alternative splicing of its transcript: HLA-G1 to HLA-G4 are membrane bound, while HLA-G5 to HLA-G7 are soluble proteins (12, 15). Soluble isoforms were detected in maternal–fetal circulation, amniotic fluid, all trophoblasts (16), human embryonic stem cells, human oocytes, and preimplantation embryos (17). Moreover, HLA-G expression differed during development of the blastocyst (17). Kotze et al. (18) showed sHLA-G as a marker in improving pregnancy outcome after IVF-ET with the ability to reduce multiple pregnancies (18). Soluble HLA-G has been detected in plasma or serum not only from pregnant women but also from non-pregnant ones; however, the concentration of sHLA-G was higher in blood from pregnant than that from non-pregnant women. In addition, sHLA-G levels were higher in the first trimester of pregnancy compared to the second and third trimesters (19), while low levels of sHLA-G have been detected in recurrent miscarriage (20), miscarriage in IVF pregnancies (21), as well as preeclampsia (22). In addition, HLA-G as an important mediator of immune escape has been also described in other immune-mediated disorders, malignancies, and transplantation (23–27).

The level of sHLA-G likely results from the *HLA-G* genotype (28, 29). One of the most studied polymorphisms of the *HLA-G* gene in the reproductive disorders was the 14-bp insertion/deletion (rs371194629: c.^*^65_^*^66insATTTGTTCATGCCT) at 3′ untranslated region (3′UTR) of exon 8. The presence of the 14-bp insertion generates an additional splice whereby 92 bases are removed from the start of exon 8 (30). Insertion of 14 bp at the 3′UTR may affect HLA-G messenger RNA stability (28), which is associated with lower levels or absence of sHLA-G in plasma (29, 31, 32). Moreover, the 14-bp insertion allele has been associated with unexplained recurrent miscarriage; however, studies have shown unclear results (33–37). According to the hypothesis of Castelli et al., HLA-G expression is determined by the combination of multiple single nucleotide polymorphisms (SNPs) (38, 39). Therefore, in addition to 14-bp insertion/deletion in 3′UTR, we decided to include in our study other polymorphic positions in the *HLA-G* gene promoter, which could affect the level of HLA-G expression, rs1632947: c.-964G>A and rs1233334: c.-725G>C/T, and to correlate them with the level of soluble HLA-G secreted into the plasma of patients measured before and after IVF-ET. These polymorphisms may affect DNA methylation. The presence of an adenine at −964 position (CpA dinucleotide) destroys a CpG dinucleotide which might be methylated (40), and the G variant at position −725 (C>G,T) that creates a CpG dinucleotide influencing on transcriptional activity of the gene (41). However, studies performed by the same group in 2006 revealed significantly higher expression level of the promoter haplotype containing the −725G allele compared with those containing the −725C or −725T alleles (42).

The aim of this study was the evaluation of sHLA-G role and its gene polymorphism in the success of implantation after IVF-ET in different clinical (fresh or frozen/thawed cycle; short or long ovarian stimulation with gonadotropin-releasing hormone GnRH antagonists or agonists) protocols.

## Materials and Methods

### Study Design

**Figure 1:**
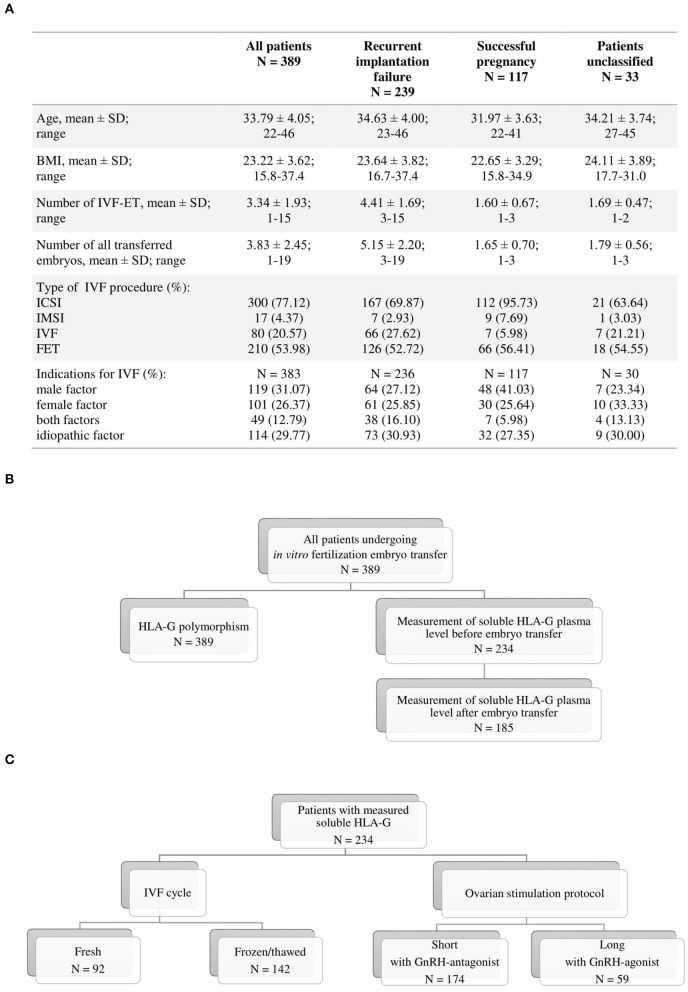
Flowchart of study population. **(A)** Characteristics of patients; **(B)** strategy of study; **(C)** division of patients by type of IVF cycle and ovarian stimulation protocol. BMI, body mass index (kg/m^2^); IVF-ET, *in vitro* fertilization–embryo transfer; ICSI, intracytoplasmic sperm injection; IMSI, intracytoplasmic morphologically selected sperm injection; FET, frozen/thawed embryo transfer; GnRH, gonadotropin-releasing hormone.

Three hundred eighty-nine patients undergoing IVF-ETs were recruited from the Department of Reproductive Medicine in Gameta Hospital, and the Department of Surgical, Endoscopic and Oncologic Gynecology, Department of Gynecology and Gynecologic Oncology, Polish Mothers' Memorial Hospital–Research Institute. In total, our group of patients underwent 1,293 embryo transfers with a total of 1,477 embryos were transferred. Among the patient group, 239 women were RIF (mean of four unsuccessful transfers) and 117 women with an ongoing pregnancy or with a child born after IVF-ET (SIVF; mean, one to two transfers). Thirty-three patients could not be qualified for RIF or SIVF because they did not maintain pregnancy after the first or second embryo transfer. Indication for IVF due to only male factors was for 119 patients (31.07%), while female factors was 101 patients (26.37%). Forty-nine patients (12.79%) were infertile due to both factors, whereas idiopathic infertility was found in 114 patients (29.77%). Patients with endometriosis were qualified for the female factor. We had no information about indications for IVF in the case of six patients. RIF and SIVF groups differed in age (*p* < 0.0001), in number of IVF embryo transfers (*p* < 0.0001), in total number of transferred embryos (*p* < 0.0001), and also in the kind of applied procedure of IVF (*p* < 0.0001). These groups also differed in factors causing their infertility that were indications for IVF (*p* = 0.0098). Only BMI (kg/m^2^) was similar in patients belonging to these groups. Detailed characteristics of patients are shown in Figure 1A, whereas strategy for the study was shown in Figure 1B.

The control fertile group was recruited from the 1st Department of Obstetrics and Gynecology, Medical University of Warsaw and from the District Hospital Strzelce Opolskie. This group consisted of 320 healthy women with at least one healthy-born child after natural conception and no history of miscarriage or endocrinological or immunological diseases: women had a mean age 32.36 years ± 5.80 (age range, 22–68). Experiments were approved by Local Ethics Committees (the agreement of Medical University of Wroclaw and Polish Mothers' Memorial Hospital–Research Institute in Lodz). Informed consent was obtained from all women included in the study.

#### Ovarian Stimulation

To stimulate ovulation, the long agonist protocol or short antagonist protocol was used. The recombinant follicle-stimulating hormone or human menopausal gonadotropin was administrated at a daily dose of 150–300 IU. In the case of the long protocol, the pituitary desensibilization was achieved by the daily administration of GnRH agonist. The growth of follicles was monitored with a transvaginal ultrasound examination and by measuring the serum level of estradiol. In female patients qualified for the short antagonist protocol, the procedure of ovarian stimulation was started on the second day of the cycle. When the mean diameter of one of the follicles was bigger than 14 mm or when the estradiol level was above 400 pg/ml, patients were administered 0.25 mg of Ganirelix acetate. After the diameter of the follicles was above 17 mm and the estradiol level was above 200 pg/ml per one follicle, patients were provided subcutaneously 250 μg recombinant chorionic gonadotropin (rhCG). Ovarian pickup was performed general intravenous anesthesia 36 h following the injection of rhCG. To supplement the luteal phase, patients were intravaginally given 2 × 200 mg micronized progesterone and orally dydrogesterone 3 × 10 mg.

#### Fertilization

After retrieval, oocytes were isolated from follicular fluid, rinsed in G-MOPS Plus (Vitrolife), and placed in G-IVF Plus (Vitrolife). About 2 h after collection, oocytes were denuded (hyaluronidase in flushing medium, Ferti Pro) and classified according to their maturity. Three to four hours after follicular aspiration, metaphase II oocytes were fertilized and individually placed in microdroplet culture dishes (Vitrolife), in 20 μl droplets of G1-PLUS (Vitrolife). Incubation was performed at 37°C, 6% CO_2_, 5% O_2_. On the following day, oocyte fertilization was checked according to zygote scoring system of Scott. On days 2 and 3, embryos were evaluated based on the number and size (even or uneven) of blastomeres, fragmentation, and multinucleation. Embryos were transferred on the third (fresh cycle) or cryopreserved on the fifth or sixth day of culture according to the age of the patient and embryo quality. Embryos underwent the cryopreservation procedure in the blastocyst stage with vitrification (Cryotop Safety Kit, Kitazato, Japan). All patients had the best quality embryos transferred. Embryo transfer was deferred in case of premature progesterone increase (>1.4 ng/ml on the day of hCG administration) or risk of ovarian hyperstimulation syndrome.

#### Endometrial Preparation and Frozen Embryo Transfer

Estradiol was administered orally, starting on the second day of the target cycle with a dosage of 6 mg/day for endometrial preparation. An ultrasound examination was performed to assess endometrial thickness and pattern 10–12 days following estradiol initiation. Endometrial thickness was measured and recorded as the maximum distance (mm) between the myometrium and endometrial surface. A thickness of ≥7.5 mm was considered satisfactory for initiation progesterone supplementation. To supplement the luteal phase, patients were intravaginal administered 3 × 200 mg micronized progesterone and orally dydrogesterone 3 × 10 mg. Thawed blastocysts were transferred on the day 5 or 6 following progesterone administration (depending developmental stage).

### DNA Preparation and Genotyping

Genomic DNA was isolated from venous blood using the Invisorb Spin Blood Midi Kit (Invitek, Berlin, Germany) according to the manufacturer's instructions. We tested *HLA-G* polymorphism in three positions: rs1632947: c.-964G>A, rs1233334: c.-725G>C/T in promoter, and rs371194629: c.^*^65_^*^66insATTTGTTCATGCCT in 3′UTR of exon 8 (Table 1). Polymorphisms in *HLA-G* gene were performed in all qualified patients by PCR-SSP method or using TaqMan assays in Real-Time PCR according to Bylinska et al. (46).

**Table 1:** Summary of tested single nucleotide polymorphisms (SNPs).

**Chr**	**Gene**	**dbSNP ID**	**Position (bp)**	**Accession number**	**Reference sequence**	**Functional region**	**Potential effect (reference)**
6	*HLA-G*	rs1632947	29826881	NC_000006.12	XM_017010817.1:c.-964G>A	Promoter	Gene expression (39, 40)
6	*HLA-G*	rs1233334	29827120	NC_000006.12	XM_005249055.1:c.-725G>C XM_005249055.1:c.-725G>T	Promoter	Gene expression (28, 32, 41, 42)
6	*HLA-G*	rs371194629	29830804-29830805	NC_000006.12	NM_002127.5: c.*65_*66 insATTTGTTCATGCCT	3′UTR of exon 8	Messenger RNA (mRNA) stability; splicing; microRNA targeting (30, 39); the 14-bp insertion allele associated with lower concentration of soluble HLA-G (28)

Genomic position is shown relative to GRCh38.p7; SNP IDs are according to dbSNP (rs, http://www.ncbi.nlm.nih.gov/SNP); c.*65_*66insATTTGTTCATGCCT was earlier described as 14-bp ins/del in 3′UTR of the HLA-G gene and were relative to the translation start site (43–45). Chr, chromosome.

### sHLA-G Measurement

We had access to 234 plasma samples taken from patients before IVF-ET, and 185 of them gave plasma samples also after IVF-ET during testing of the beta-subunit of human chorionic gonadotropin (Figure 1B). Among this group of patients, 92 (39.32%) underwent fresh cycles. One hundred forty-two patients (60.68%) underwent frozen/thawed cycles. In addition, 59 patients (25.32%) underwent a long ovarian stimulation protocol with GnRH agonist, and 174 patients (74.68%) underwent a short protocol with GnRH antagonist (Figure 1C).

Plasma samples were stored at −80°C until the time of assay. The concentration of sHLA-G (IU/ml) in plasma of patients before and after IVF-ET was tested with a sandwich enzyme-linked immunosorbent assay (ELISA) kit following the manufacturer's protocol (Exbio/Biovendor, Czech Republic). Standard curve measured the concentration of HLA-G1 (which are shed from the cell surface by proteolytic cleavage) and sHLA-G5 isoforms from 3.91 to 125 IU/ml. The limit of sHLA-G detection in this test was 0.6 IU/ml. Samples in which the sHLA-G concentration exceeded 125 IU/ml were retested after diluting them by a factor 1:8. Samples in which no sHLA-G was detected were also once again analyzed.

### Statistical Analysis

For the analysis of allelic and genotypic frequencies of HLA-G, we used the two-tailed Fisher exact test (GraphPad Prism 5 software). Hardy–Weinberg equilibrium was estimated using the chi-square test with 1 df. All genotype frequencies were in Hardy–Weinberg equilibrium both in control and in patient groups. A *p* < 0.05 was considered significant. The odds ratio (OR) and its 95% CI were computed as the measure of effect size. For multiple comparison tests, the Bonferroni correction was done. Haplotypes were generated by FAMHAP (http://famhap.meb.uni-bonn.de). Statistical analyses concerning sHLA-G concentration measured in the plasma of patients before and after embryo transfer were performed using Mann–Whitney and Spearman rank correlation test (GraphPad Prism 5 software). To identify a cut-off level of sHLA-G suggestive of likelihood of getting pregnancy, receiver–operator curve analysis was performed (GraphPad Prism 5 software). All parameters of statistical analyses (numbers, medians, means, standard deviation and errors, min, max, and 25–75% percentiles) are part of Supplementary Tables 1–12. When, as a result of statistical analysis, no conclusions could be drawn due to the insufficient number of patients, e.g., for most diplotype-related analyses, these results were not discussed in *Results*, but the data can be viewed in the following Supplementary Tables 6–8, 10, 12.

## Results

### *HLA-G* Genotypes, Haplotypes, and Diplotypes Association With Infertility and RIF

We found significant differences in genotype frequencies between patients and fertile women only in rs1632947: c.-964G>A. The GG genotype was more prevalent in fertile women than patients undergoing IVF-ET (*p* = 0.025, OR = 0.61), RIF patients (*p* = 0.07, OR = 0.64), and SIVF women (*p* = 0.01, OR = 0.43). However, minor allele A frequency was more represented in all patients (*p* = 0.022, OR = 1.28), RIF (*p* = 0.07, OR = 1.25) and SIVF women (*p* = 0.009, OR = 1.49) than in fertile women (Table 2).

**Table 2:** *HLA-G* genotype and minor allele frequencies in women from control and patient groups.

**Genotype**	**Control (%)**	**All patients (%)**	**RIF (%)**	**SIVF (%)**	**All patients vs. Control**			**RIF vs. control**			**SIVF vs. control**			**RIF vs. SIVF**		
					***p-*value**	**OR**	**95% CI**	***p-*value**	**OR**	**95% CI**	***p-*value**	**OR**	**95% CI**	***p-*value**	**OR**	**95% CI**
rs1632947:−964G>A	*N* = 320	*N* = 389	*N* = 239	*N* = 117												
AA^*^	64 (20.00)	104 (26.74)	64 (26.78)	33 (28.21)		1			1			1			1	
AG	161 (50.31)	191 (49.10)	114 (47.70)	63 (53.85)	0.11	0.73	0.50–1.06	0.13	0.71	0.46–1.08	0.29	0.76	0.46–1.27	0.89	0.93	0.55–1.57
GG	95 (29.69)	94 (24.16)	61 (25.52)	21 (17.94)	**0.025**	**0.61**	**0.40**–**0.93**	0.07	0.64	0.40–1.03	**0.01**	**0.43**	**0.23**–**0.81**	0.25	1.50	0.78–2.87
Minor allele A	289 (45.16)	399 (51.29)	242 (50.63)	129 (55.13)	**0.022**	**1.28**	**1.04**–**1.58**	0.07	1.25	0.98–1.58	**0.009**	**1.49**	**1.10**–**2.02**	0.26	0.83	0.61–1.14
H–W	0.78	0.73	0.48	0.34												
rs1233334:−725 G>C/T	*N* = 320	*N* = 389	*N* = 239	*N* = 117												
CC^*^	220 (68.75)	274 (70.44)	173 (72.38)	78 (66.67)		1			1			1			1	
CG	79 (24.69)	89 (22.88)	52 (21.76)	28 (23.94)	0.59	0.90	0.64–1.29	0.42	0.84	0.56–1.25	1.00	1.00	0.65–1.65	0.58	0.84	0.49–1.43
GG	8 (2.50)	5 (1.29)	4 (1.67)	1 (0.85)	0.27	0.50	0.16–1.56	0.56	0.64	0.19–2.15	0.46	0.35	0.04–2.87	1.00	1.80	0.20–16.41
GT	1 (0.31)	3 (0.78)	2 (0.84)	1 (0.85)	0.63	2.41	0.25–23.33	0.59	2.54	0.23–28.30	0.46	2.82	0.17–45.67	1.00	0.90	0.08–10.10
CT	12 (3.75)	18 (4.63)	8 (3.35)	9 (7.69)	0.71	1.20	0.57–2.55	0.82	0.85	0.34–2.12	0.13	2.12	0.86–5.22	0.10	0.40	0.15–1.08
TT	0 (0.00)	0 (0.00)	0 (0.00)	0 (0.00)	–	–	–	–	–	–	–	–	–	–	–	–
Minor allele T	13 (2.03)	21 (2.70)	10 (2.09)	10 (4.27)	0.49	1.34	0.66–2.69	1.00	1.03	0.45–2.37	0.09	2.15	0.93–4.98	0.14	0.48	0.20–1.13
H–W	0.91	0.51	0.34	0.88												
rs371194629: ins ATTTGTTCATGCCT/del	*N* = 320	*N* = 389	*N* = 239	*N* = 117												
Del/del^*^	115 (35.94)	128 (32.90)	80 (33.47)	37 (31.62)		1			1			1			1	
Ins/del	153 (47.81)	197 (50.64)	121 (50.63)	61 (52.14)	0.40	1.16	0.83–1.61	0.51	1.14	0.78–1.65	0.40	1.24	0.77–1.99	0.80	0.92	0.56–1.51
Ins/ins	52 (16.25)	64 (16.45)	38 (15.90)	19 (16.24)	0.73	1.11	0.71–1.72	0.90	1.05	0.63–1.74	0.74	1.14	0.60–2.16	0.86	0.93	0.47–1.82
Minor allele ins	257 (40.16)	325 (41.77)	197 (41.21)	99 (42.31)	0.55	1.07	0.86–1.32	0.76	1.05	0.82–1.33	0.59	1.09	0.81–1.48	0.81	0.96	0.70–1.31
H–W	0.93	0.42	0.49	0.46												

*Values in bold indicate significant differences. RIF, recurrent implantation failure; SIVF, successful IVF; H–W, Hardy–Weinberg equilibrium; P, probability; OR, odds ratio; 95% CI, 95% confidence interval from two-sided Fisher's exact test*.

* *Reference*.

Among controls and patients, 10 haplotypes in the following order rs1632947–rs1233334–rs371194629 were generated. Comparison of haplotype frequencies between patients and fertile controls revealed a highly significant association of A-C-del (*p*_corr_ < 0.001), A-G-del (*p*_corr_ < 0.001), and G-C-ins (*p*_corr_ < 0.001) haplotypes with infertility, while G-C-del (*p*_corr_ < 0.001), G-G-del (*p*_corr_ = 0.027), and A-C-ins (*p*_corr_ = 0.022) haplotypes with protection against disease (Table 3). Similar results (in the same direction) for above-mentioned haplotypes were obtained when RIF and SIVF patients were compared with fertile women. In addition, haplotype frequencies of patients with successful pregnancies after IVF exhibited a similar distribution to RIF patients except for G-C-del haplotype, which was significantly more frequent in RIF patients. However, after correction for multiple comparisons, this significance was lost (*p* = 0.05, *p*_corr_ = ns; Table 3).

**Table 3:** *HLA-G* haplotype frequencies in women from control and patient groups.

Haplotype*	**Control (%) 2*N* = 640**	**All patients (%) 2*N* = 778**	**RIF (%) 2*N* = 478**	**SIVF (%) 2*N* = 234**	**All Patients vs. control**				**RIF vs. control**				**SIVF vs. control**				**RIF vs. SIVF**			
					***p***	**OR**	**95% CI**	***p*_**corr.**_**	***p***	**OR**	**95% CI**	***p*_**corr.**_**	***p***	**OR**	**95% CI**	***p*_**corr.**_**	***p***	**OR**	**95% CI**	***p*_**corr.**_**
A C del	46 (7.19)	132 (16.97)	72 (15.06)	47 (20.09)	**<0.0001**	**2.64**	**1.85–3.76**	**<0.001**	**<0.0001**	**2.29**	**1.55–3.39**	**<0.001**	**<0.0001**	**3.25**	**2.09–5.03**	**<0.001**	0.11	0.71	0.47–1.06	–
A C ins	241 (37.66)	232 (29.82)	154 (32.22)	66 (28.21)	**0.0022**	**0.70**	**0.56**–**0.88**	**0.022**	0.067	0.79	0.61–1.01	–	**0.01**	**0.65**	**0.47**–**0.91**	0.1	0.30	1.21	0.86–1.71	–
A G del	0 (0.00)	29 (3.73)	14 (2.93)	12 (5.13)	**<0.0001**	**50.42**	**3.07**–**827.38**	**<0.001**	**<0.0001**	**39.99**	**2.34**–**672.50**	**<0.001**	**<0.0001**	**71.97**	**4.24**–**1221**	**<0.001**	0.14	0.56	0.25–1.23	–
A T del	2 (0.31)	5 (0.64)	2 (0.42)	3 (1.28)	0.47	2.06	0.40–10.68	–	1.00	1.34	0.19–9.55	–	0.12	4.14	0.69–24.96	–	0.34	0.32	0.05–1.95	–
A T ins	0 (0.00)	1 (0.13)	0 (0.00)	1 (0.43)	1.00	2.47	0.10–60.82	–	–	–	–	–	0.27	8.23	0.33–202.87	–	0.33	0.16	0.007–4.01	–
G C del	242 (37.81)	215 (27.63)	145 (30.33)	54 (23.08)	**<0.0001**	**0.63**	**0.50**–**0.79**	**<0.001**	**0.011**	**0.72**	**0.56**–**0.92**	0.11	**<0.0001**	**0.49**	**0.35**–**0.70**	**<0.001**	**0.05**	**1.45**	**1.01**–**2.08**	0.5
G C ins	2 (0.31)	76 (9.77)	35 (7.32)	26 (11.11)	**<0.0001**	**34.54**	**8.45**–**141.24**	**<0.001**	**<0.0001**	**25.20**	**6.03**–**105.36**	**<0.001**	**<0.0001**	**39.88**	**9.38**–**169.49**	**<0.001**	0.12	0.63	0.37–1.08	–
G G del	93 (14.53)	72 (9.25)	48 (10.04)	19 (8.12)	**0.0027**	**0.60**	**0.43**–**0.83**	**0.027**	**0.029**	**0.66**	**0.45**–**0.95**	0.29	**0.012**	**0.52**	**0.31**–**0.87**	0.12	0.49	1.26	0.72–2.20	–
G G ins	3 (0.47)	1 (0.13)	0 (0.00)	0 (0.00)	0.33	0.27	0.02–2.64	–	0.27	0.19	0.010–3.70	–	0.57	0.39	0.020–7.55	–	–	–	–	–
G T ins	11 (1.72)	15 (1.93)	8 (1.67)	6 (2.56)	0.84	1.12	0.51–2.47	–	1.00	0.97	0.39–2.44	–	0.41	1.51	0.55–4.12	–	0.41	0.65	0.22–1.89	–

Values in bold indicate significant differences. RIF, recurrent implantation failure; SIVF, successful IVF; p, probability; p_corr._, p × 10 possible haplotypes—Bonferroni correction; OR, odds ratio; 95% CI, 95% confidence interval from two-sided Fisher's exact test; Haplotypes* were estimated in the following order: rs1632947:−964G>A; rs1233334:−725G>C/T; rs371194629:insATTTGTTCATGCCT/del.

We also estimated 23 different diplotypes: 19 of them were present in the control group, while 22 were present in IVF patients. To determine the diplotypes, we adopted the same order of tested SNPs as in the case of haplotypes (Table 4). For nine diplotypes, we found differences in frequencies between patients and controls. Diplotypes A-C-del/A-C-del, A-C-del/A-G-del, G-C-del/G-C-ins, and G-C-ins/G-C-ins were more prevalent in patients than controls (*p*_corr_ < 0.0023). These diplotypes were absent or extremely rare in fertile women, whereas five diplotypes were protective and present in higher frequencies in the control group. Comparison between patients and controls revealed significant differences for A-C-ins/A-C-ins (*p* = 0.0087, *p*_corr_ = ns), A-G-del/A-G-del (*p* = 0.041, *p*_corr_ = ns), G-C-del/G-C-del (*p*_corr_ = 0.0069), G-G-del/G-C-del (*p*_corr_ = 0.0092), and G-G-del/G-G-del (*p* = 0.026, *p*_corr_ = ns) diplotypes (Table 4). When we divided the patient group into RIF and SIVF and compared them with control women, we found interesting observations. Namely, A-C-del/A-C-del, A-C-del/A-G-del, and G-C-ins/G-C-ins diplotypes were more prevalent in RIF and SIVF than in controls (all comparisons *p*_corr_ < 0.0023). Less significance was achieved for comparison of G-C-del/G-C-ins positive patients (*p*_corr_ < 0.0023 for RIF and *p*_corr_ = 0.044 for SIVF). When we compared diplotypes which differed only in the insertion allele (G-C-del/G-C-del vs. G-C-del/G-C-ins vs. G-C-ins/G-C-ins, Kruskal–Wallis test), we observed a very significant association with infertility expressed by higher frequency in all patients, RIF, and also SIVF (*p* < 0.0001 for comparisons with fertile control, Table 4). Insertion allele in these diplotypes was disadvantageous because odds ratios increased from protective 0.38 in G-C-del/G-C-del women to predisposing 18.20 in G-C-del/G-C-ins women and 48.63 in G-C-ins/G-C-ins women. However, the insertion allele in diplotypes A-C-ins/A-C-del and A-C-ins/A-C-ins was protective, and odds ratios ranged from 28.59 in A-C-del/A-C-del patients to 0.61 in A-C-ins/A-C-del and 0.51 in A-C-ins/A-C-ins patients (Table 4). We can conclude that *HLA-G* polymorphism is associated with infertility.

**Table 4:** *HLA-G* diplotype frequencies in women from control and patient groups.

Diplotype^*^	**Control (%) *N* = 320**	**All patients (%) *N* = 389**	**RIF (%) *N* = 239**	**SIVF (%) *N* = 117**	**All patients vs. control**				**RIF vs. control**				**SIVF vs. control**				**RIF vs. SIVF**			
					***p***	**OR**	**95% CI**	***p_***corr*.**_***	***p***	**OR**	**95% CI**	***p*_**corr.**_**	***p***	**OR**	**95% CI**	***p_***corr*.**_***	***p***	**OR**	**95% CI**	***p_***corr*.**_***
A C del/A C del	1 (0.31)	32 (8.23)	16 (6.69)	12 (10.26)	**<0.0001**	**28.59**	**3.88–210.56**	**0.0023**	**<0.0001**	**22.13**	**2.92–167.55**	**<0.0023**	**<0.0001**	**34.54**	**4.46–267.29**	**<0.0023**	0.30	0.64	0.30–1.38	–
A C del/A G del	0 (0.00)	20 (5.14)	8 (3.35)	9 (7.69)	**<0.0001**	**35.56**	**2.14**–**590.75**	**0.0023**	**0.0011**	**23.14**	**1.33**–**402.23**	**0.025**	**<0.0001**	**53.97**	**3.13**–**931.63**	**<0.0023**	0.11	0.43	0.16–1.12	–
A C del/G C del	20 (6.25)	21 (5.40)	12 (5.02)	8 (6.84)	0.63	0.86	0.46–1.61	–	0.59	0.80	0.39–1.65	–	0.83	1.10	0.48–2.53	–	0.48	0.73	0.29–1.81	–
A C del/G G del	5 (1.56)	10 (2.57)	7 (2.93)	3 (2.56)	0.44	1.66	0.56–4.92	–	0.38	1.89	0.60–5.99	–	0.45	1.65	0.39–6.96	–	1.00	1.14	0.29–4.47	–
A C ins/A C del	17 (5.31)	13 (3.34)	10 (4.18)	3 (2.56)	0.26	0.61	0.29–1.29	–	0.69	0.78	0.36–1.73	–	0.31	0.48	0.14–1.64	–	0.56	1.66	0.45–6.15	–
A C ins/A C ins	44 (13.75)	29 (7.46)	25 (10.46)	4 (3.42)	**0.0087**	**0.51**	**0.31**–**0.83**	ns	0.31	0.75	0.45–1.24	–	**0.0021**	**0.24**	**0.08**–**0.66**	**0.048**	**0.026**	**3.17**	**1.09**–**9.23**	ns
A C ins/A T del	1 (0.31)	4 (1.03)	1 (0.42)	3 (2.56)	0.39	3.31	0.37–29.82	–	1.00	1.34	0.08–21.49	–	0.06	8.30	0.86–80.22	–	0.11	0.16	0.02–1.56	–
A C ins/G C del	92 (28.75)	108 (27.76)	67 (28.03)	32 (27.35)	0.81	0.95	0.69–1.32	–	0.93	0.97	0.69–1.36	–	0.83	0.94	0.62–1.46	–	1.00	1.03	0.65–1.62	–
A G del/A G del	0 (0.00)	4 (1.03)	3 (1.26)	1 (0.85)	**0.041**	**0.09**	**0.005**–**1.68**	ns	0.08	9.43	0.49–183.11	–	0.27	8.23	0.33–202.87	–	1.00	1.47	0.15–14.232	–
A G del/A T ins	0 (0.00)	1 (0.26)	0 (0.00)	1 (0.85)	1.00	2.48	0.10–61.01	–	–	–	–	–	0.27	8.23	0.33–202.87	–	0.33	0.16	0.007–4.01	–
A T del/A C del	1 (0.31)	1 (0.26)	1 (0.42)	0 (0.00)	1.00	0.82	0.05–13.21	–	1.00	1.34	0.08–21.49	–	1.00	0.91	0.04–22.41	–	1.00	1.47	0.06–36.33	–
G C del/G C del	45 (14.06)	23 (5.91)	19 (7.95)	3 (2.56)	**0.0003**	**0.38**	**0.23**–**0.65**	**0.0069**	**0.037**	**0.55**	**0.32**–**0.95**	ns	**0.0004**	**0.17**	**0.05**–**0.56**	**0.0092**	0.06	3.19	10.89	–
G C del/G C ins	1 (0.31)	21 (5.40)	13 (5.44)	6 (5.13)	**<0.0001**	**18.20**	**2.43**–**136.16**	**0.0023**	**0.0001**	**17.87**	**2.33**–**137.11**	**0.0023**	**0.0019**	**16.82**	**2.01**–**140.50**	**0.044**	1.00	1.06	0.40–2.83	–
G C ins/G C ins	0 (0.00)	27 (6.94)	11 (4.6)	10 (8.55)	**<0.0001**	**48.63**	**2.95**–**800.96**	**0.0023**	**<0.0001**	**31.51**	**1.85**–**536.45**	**<0.0023**	**<0.0001**	**59.91**	**3.49**–**1027.3**	**<0.0023**	0.16	0.53	0.22–1.26	–
G C ins/G G ins	1 (0.31)	1 (0.26)	0 (0.00)	0 (0.00)	1.00	0.82	0.05–13.21	–	1.00	0.45	0.02–10.97	–	1.00	0.91	0.04–22.41	–	–	–	–	–
G G del/A C ins	37 (11.56)	42 (10.80)	24 (10.04)	15 (12.82)	0.81	0.93	0.58–1.48	–	0.60	0.86	0.51–1.46	–	0.75	1.12	0.60–2.07	–	0.48	0.77	0.37–1.50	–
G G del/G C del	36 (11.25)	16 (4.11)	13 (5.44)	1 (0.85)	**0.0004**	**0.34**	**0.18**–**0.62**	**0.0092**	**0.019**	**0.47**	**0.25**–**0.89**	ns	**0.0002**	**0.07**	**0.01**–**0.53**	**0.0046**	**0.04**	**6.51**	**0.85**–**50.12**	ns
G G del/G G del	7 (2.19)	1 (0.26)	1 (0.42)	0 (0.00)	**0.026**	**0.12**	**0.01**–**0.94**	ns	0.15	0.19	0.02–1.55	–	0.20	0.18	0.01–3.17	–	1.00	1.47	0.06–36.33	–
G G ins/G G ins	1 (0.31)	0 (0.00)	0 (0.00)	0 (0.00)	0.45	0.27	0.01–6.74	–	1.00	0.45	0.018–10.97	–	1.00	0.91	0.04–22.41	–	–	–	–	–
G T ins/A C del	1 (0.31)	3 (0.77)	2 (0.84)	0 (0.00)	0.63	2.48	0.26–23.96	–	0.58	2.69	0.24–29.71	–	1.00	0.91	0.04–22.41	–	1.00	2.46	0.12–51.50	–
G T ins/A C ins	6 (1.88)	7 (1.80)	2 (0.84)	5 (4.27)	0.58	0.70	0.23–2.11	–	0.48	0.44	0.09–2.21	–	0.18	2.31	0.70–7.64	–	0.42	0.19	0.04–1.00	–
G T ins/G C del	3 (0.94)	3 (0.77)	2 (0.84)	1 (0.85)	1.00	0.82	0.16–4.10	–	1.00	0.89	0.15–5.36	–	1.00	0.91	0.09–8.81	–	1.00	0.98	0.09–10.86	–
G T ins/G G del	1 (0.31)	2 (0.50)	2 (0.84)	0 (0.00)	1.00	1.65	0.15–18.28	–	0.58	2.69	0.24–29.71	–	1.00	0.54	0.03–11.39	–	1.00	2.46	0.12–51.50	–

Values in bold indicate significant differences. RIF, recurrent implantation failure; SIVF, successful IVF; P, probability; P corr., p ×23 possible diplotypes—Bonferroni correction; ns, not significant; OR, odds ratio; 95% CI, 95% confidence interval from two-sided Fisher's exact test.

* *Diplotypes were estimated in the following order: rs1632947:−964G>A; rs1233334:−725G>C/T; rs371194629:insATTTGTTCATGCCT/del; comparison of diplotypes G C del/G C del, G C del/G C ins and G C ins/G C ins between all patients vs. control, RIF vs. control, and SIVF vs. control by Kruskal–Wallis test, p < 0.0001*.

### Impact of *HLA-G* Haplotypes on Soluble HLA-G Plasma Level and Pregnancy Outcome in Patients Undergoing IVF-ET

**Figure 2:**
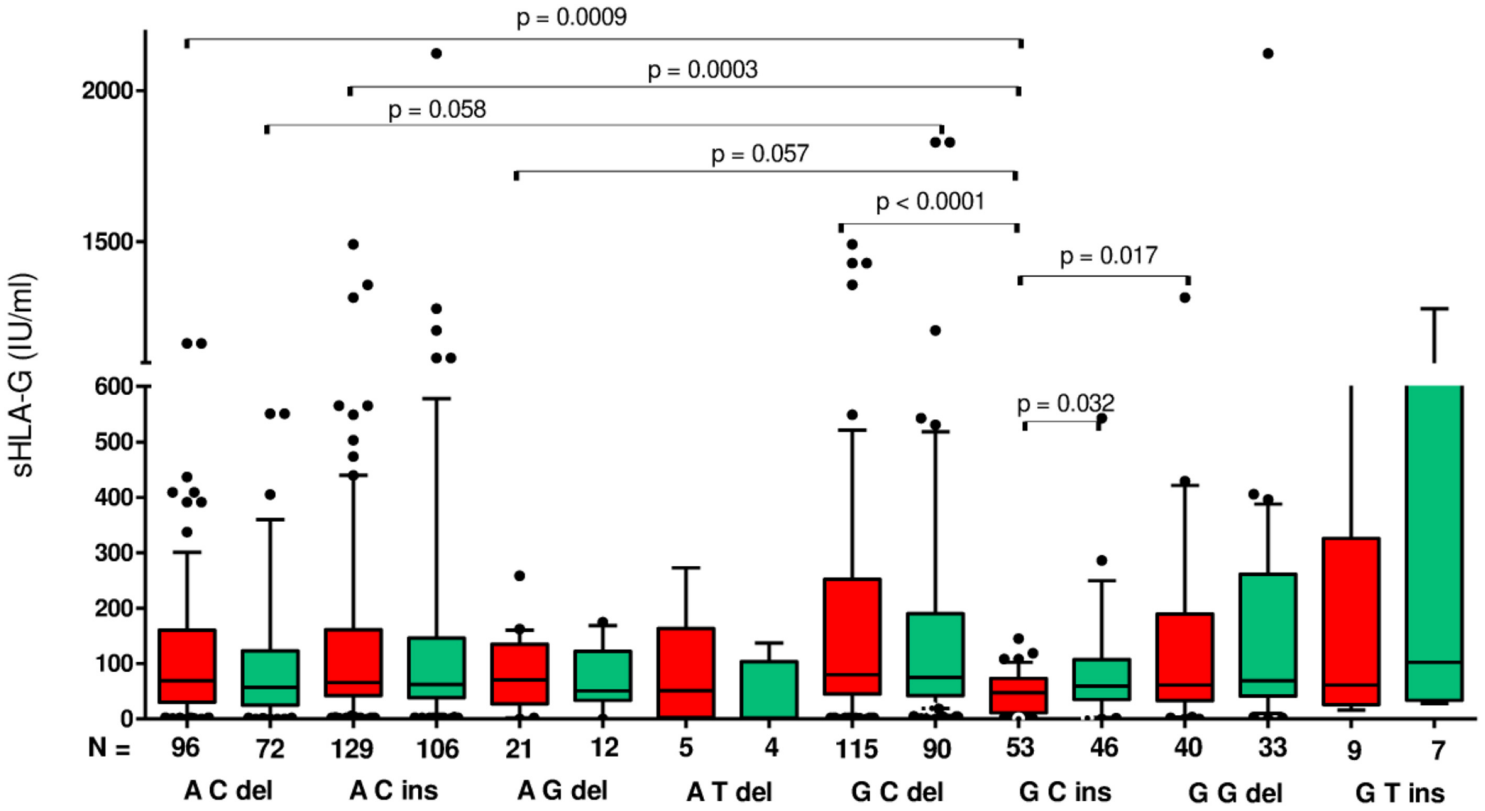
Concentration of soluble HLA-G per milliliter of plasma (IU/ml) measured before and after embryo transfer in all patients according to *HLA-G* haplotypes. Haplotypes were estimated in the following order: rs1632947:−964G>A; rs1233334:−725G>C/T; rs371194629:insATTTGTTCATGCCT/del. Red boxes represent the level of sHLA-G measured before embryo transfer and green boxes, after embryo transfer. Boxes are drawn from the first quartile (25th Percentile) to the third quartile (75th Percentile). Black lines in boxes are medians. Whiskers represent 10–90 percentiles. *N* is the number of patients. *p*-values are calculated by Mann–Whitney test.

**Figure 3:**
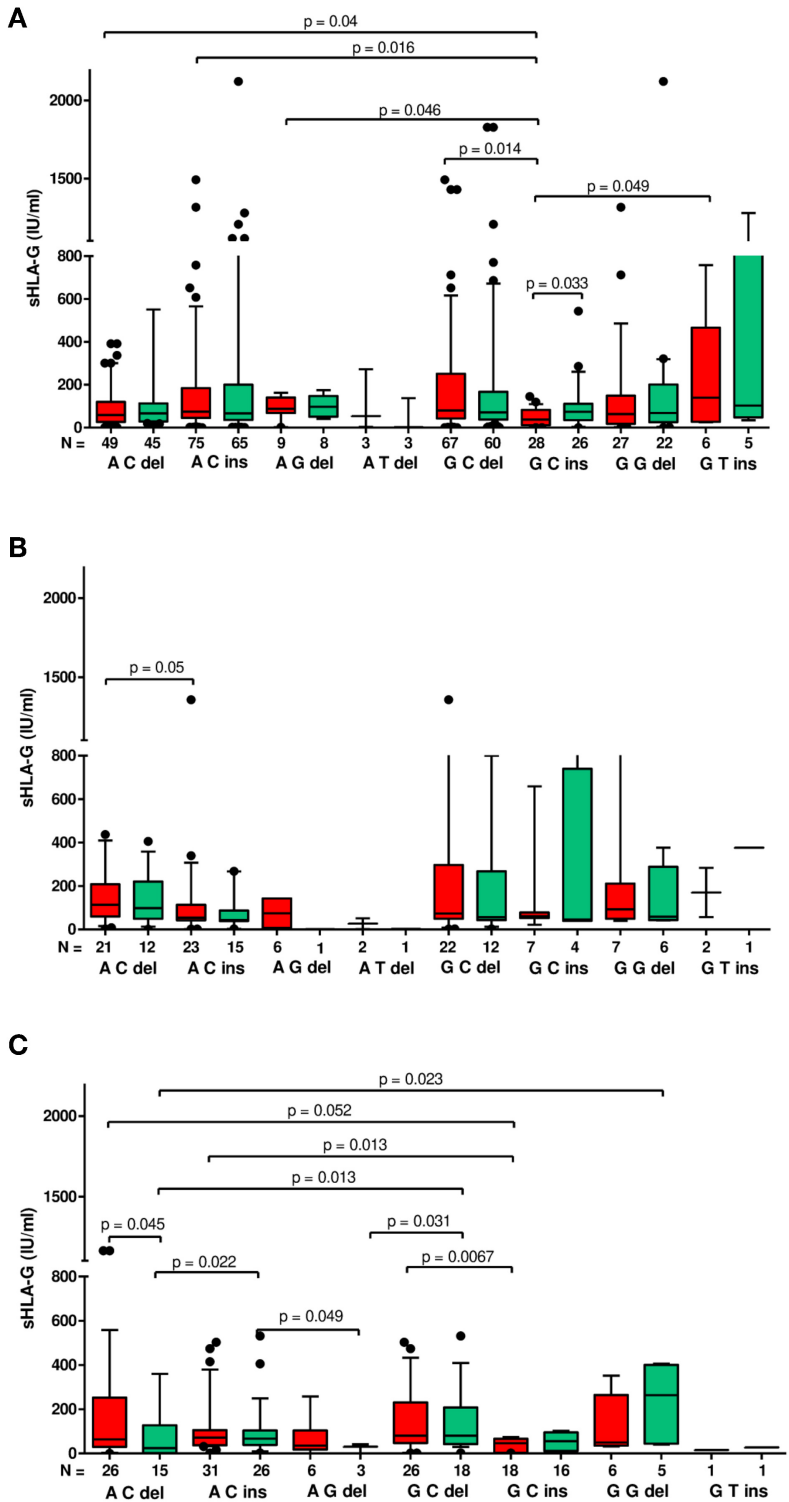
Concentration of soluble HLA-G per ml of plasma (IU/ml) according to *HLA-G* haplotypes in different clinical outcomes. Haplotypes were estimated in the following order: rs1632947:−964G>A; rs1233334:−725G>C/T; rs371194629:insATTTGTTCATGCCT/del. sHLA-G was measured in patients who achieved clinical pregnancy **(A)**, with lack of pregnancy **(B)**, and with miscarriage **(C)**. Boxes are drawn from the first quartile (25th percentile) to the third quartile (75th percentile). Black lines in boxes are medians. Whiskers represent 10–90 percentiles. *N* is the number of patients. sHLA-G was measured before (red boxes) and after embryo transfer (green boxes). *p*-values are calculated by Mann–Whitney test.

Although a very wide range of soluble HLA-G was detected in plasma patients (0–2,112 IU/ml), we found that patients carrying particular haplotypes differed in secretion of sHLA-G (Figure 2) and its influence on pregnancy outcome (Figure 3). The most striking difference in concentration of sHLA-G measured before embryo transfer was achieved in patients with G-C-del and G-C-ins haplotypes (*p* < 0.0001; median, 79.95 vs. 47.34 IU/ml, respectively; Figure 2, Supplementary Table 1). Also in patients with A-C-del (median, 69.02), A-C-ins (median, 65.72), and G-G-del (median, 61.5) haplotypes, we detected a higher level of sHLA-G than in G-C-ins patients (median, 47.34). These comparisons were very significant, *p* = 0.0009, *p* = 0.0003, *p* = 0.017, respectively. Such observations were not detected in the sHLA-G level measured after embryo transfer, probably due to the differences in pregnancy outcome.

Moreover, G-C-del carriers secreted a higher concentration of sHLA-G than G-C-ins carriers when embryo transfer resulted in a clinical pregnancy (*p* = 0.014; median, 79.95 vs. 37.21 IU/ml; Figure 3A, Supplementary Table 2). Even stronger significance (*p* = 0.0067; median, 80.77 vs. 46.76 IU/ml) was obtained when embryo transfer resulted in a miscarriage (Figure 3C, Supplementary Table 4). Secretion of sHLA-G in plasma from A-C-ins women also differed from those carrying G-C-ins when embryo transfer resulted in pregnancy (*p* = 0.016; median, 74.56 vs. 37.21 IU/ml; Figure 3A, Supplementary Table 2) and in a miscarriage (*p* = 0.013; median, 71.83 vs. 46.76; Figure 3C, Supplementary Table 4). Similar observations were obtained for A-C-del vs. G-C-ins carriers in pregnancy (*p* = 0.04; median, 58.95 vs. 37.21 IU/ml; Figure 3A, Supplementary Table 2) and in a miscarriage (*p* = 0.05; median, 64.10 vs. 46.76 IU/ml; Figure 3C, Supplementary Table 4). Less significant differences (*p* ~ 0.05) were found also for A-G-del, G-T-ins vs. G-C-ins haplotypes comparisons.

In addition, when embryo transfer resulted in pregnancy, the means and/or medians of sHLA-G concentrations were increased or were, at least, kept at the level of those measured before the embryo transfer (Supplementary Table 2). By discarding the sHLA-G extreme values, i.e., below the 25th percentile and above the 95 percentile, we were able to determine the concentration of sHLAG for which patient achieved and maintained pregnancy. We ranked these values according to haplotypes and from highest to lowest. This analysis showed that the minimum concentration of sHLA-G needed to get pregnant is 37.21 IU/ml for G-C-ins haplotype, but to maintain pregnancy, a woman should secrete at least 61.10 IU/ml (Table 5).

**Table 5:** Association of *HLA-G* haplotypes with soluble HLA-G measured before and after embryo transfer in patients with pregnancy.

**Haplotype**	**Soluble HLA-G**	
	**Before embryo transfer median (IU/ml)**	**After embryo transfer median (IU/ml)**
G-C-del	73.80	71.17
A-C-ins	67.67	66.21
G-G-del	63.38	68.44
A-C-del	58.95	61.10
G-C-ins	37.21	67.64

*Medians were calculated after rejection of extreme values, i.e., below the 25th percentile and above the 95 percentile. Haplotypes were ranked from the highest to the lowest secreting*.

When embryo transfer resulted in a lack of pregnancy (Supplementary Table 3), we observed in general a decrease in sHLA-G level (expressed by means and medians) after embryo transfer. In the third case, when embryo transfer lead to complications such as miscarriage, we found a reduction in sHLA-G in patients carrying A-C-del, A-C-ins, and A-G-del haplotypes, while in G-C-ins, G-G-del, and G-T-ins patients, we found an increase in sHLA-G level. Only in patients with G-C-del haplotypes did the sHLA-G level not change (Supplementary Table 4). This indicates that miscarriage may not be due solely to sHLA-G levels, but other factors may also affect pregnancy complications.

**Figure 4:**
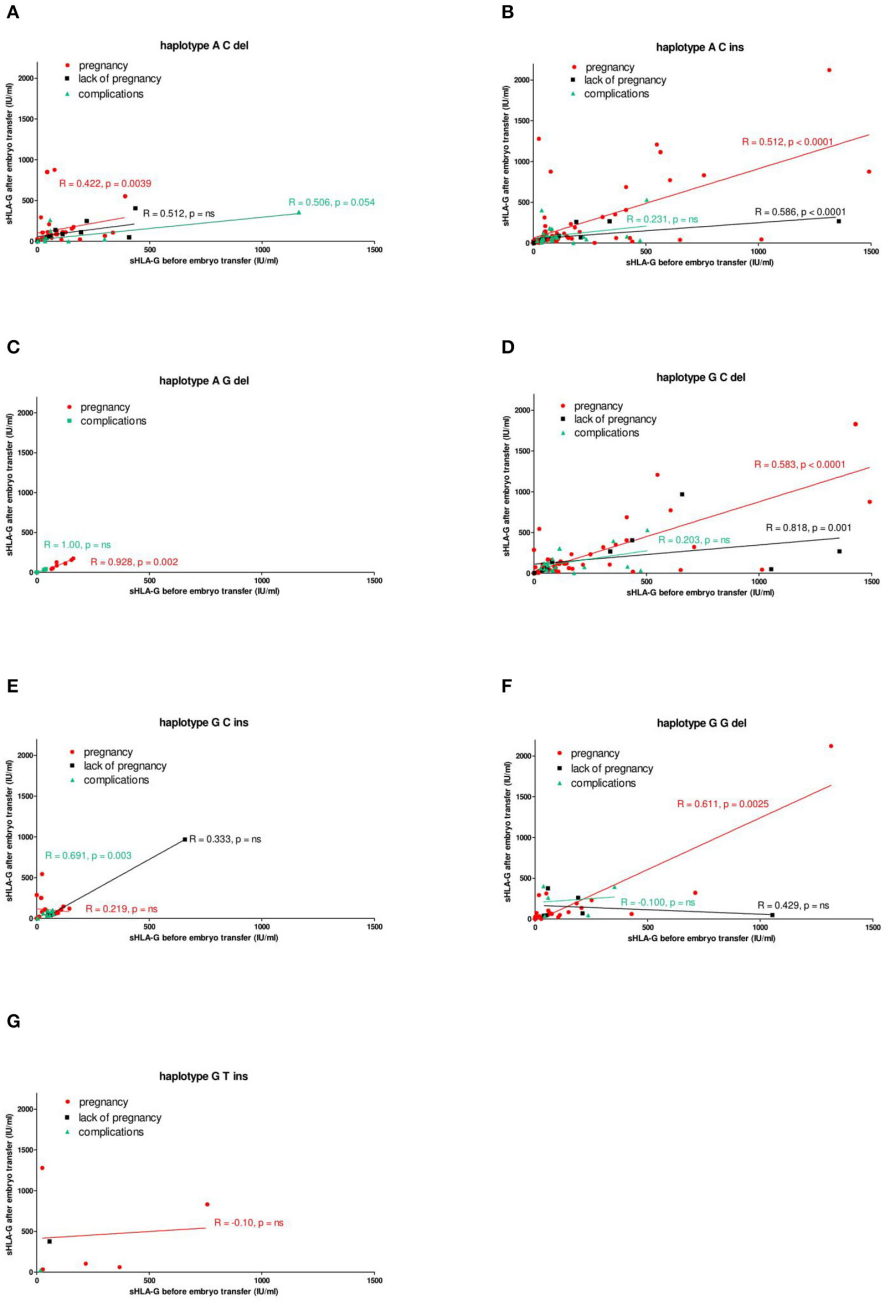
Correlation of soluble HLA-G level with pregnancy outcome, dependent on HLA-G haplotypes. Haplotypes were estimated in all patients in the following order: rs1632947:−964G>A; rs1233334:−725G>C/T; rs371194629:insATTTGTTCATGCCT/del and presented as **(A)** ACdel, **(B)** ACins, **(C)** AGdel, **(D)** GCdel, **(E)** GCins, **(F)** GGdel, and **(G)** GTins. Pregnancy was represented by red lines, lack of pregnancy by black lines, and complications (miscarriage) by green lines. Correlation was obtained with Spearman correlation test (*R*, Spearman correlation coefficient; *p, p*-value). On the *x* and *y* axes are values for sHLA-G (IU/ml) before and after embryo transfer.

Nevertheless, Spearman correlation analysis of sHLA-G secretion in particular women (Figures 4A–G) revealed positive correlation with pregnancy in almost all detected haplotypes: (Figure 4A) A-C-del (coefficient correlation *R* = 0.422, *p* = 0.0039), (Figure 4B) A-C-ins (*R* = 0.512, *p* < 0.0001), (Figure 4C) A-G-del (*R* = 0.928, *p* = 0.002), (Figure 4D) G-C-del (*R* = 0.583, *p* < 0.0001), and (Figure 4F) G-G-del (*R* = 0.611, *p* = 0.0025). Only patients carrying G-C-ins (Figure 4E) and G-T-ins (Figure 4G) haplotype did not demonstrate positive correlation with pregnancy. What is more, it is also worth noting that patients who had a miscarriage or did not become pregnant were also seen in all haplotypes. We can conclude from this part of the analysis that patients who are positive for the G-C-ins haplotype have the least chance of getting pregnant and maintaining pregnancy.

However, when we performed receiver–operator curve analysis (area under curve, 66%) for patients regardless of possessed haplotype, we found that 59.73 IU/ml sHLA-G is the threshold value with the best sensitivity (58.82%) and specificity (66.10%) to discriminate patients who achieved and maintained pregnancy from those who did not get pregnant or had a miscarriage (*p* = 0.0085; likelihood ratio, 1.74; 95% CI = 0.55–0.78).

### Impact of *HLA-G* Diplotypes on sHLA-G Plasma Level and Pregnancy Outcome in Patients Undergoing IVF-ET

**Figure 5:**
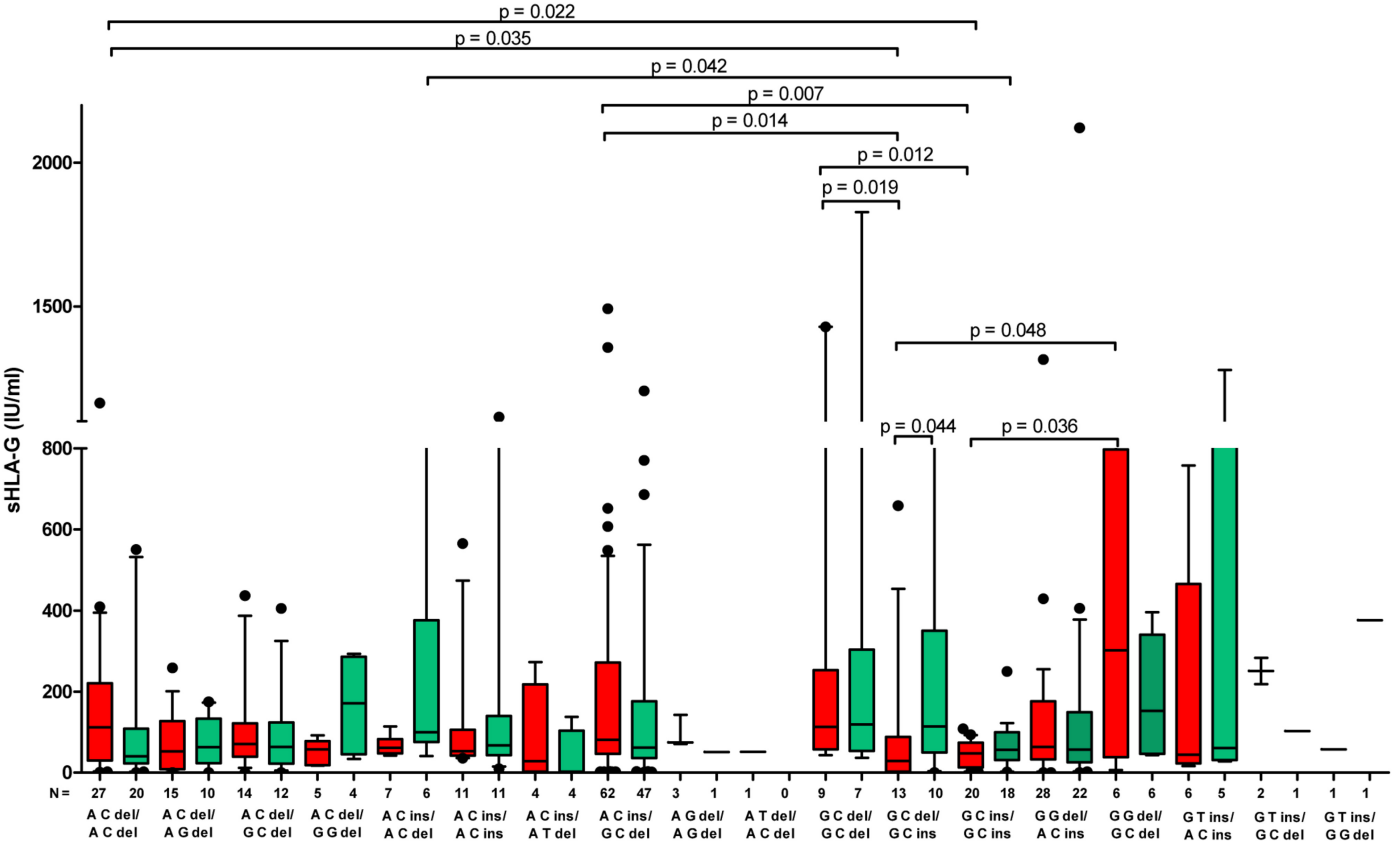
Concentration of soluble HLA-G per milliliter of plasma (IU/ml) measured before and after embryo transfer in all patients according to their *HLA-G* diplotypes. Diplotypes were determined from haplotype analysis and estimated in the following order: rs1632947:−964G>A; rs1233334:−725G>C/T; rs371194629:insATTTGTTCATGCCT/del. Red boxes represent the level of sHLA-G measured before embryo transfer and green boxes, after embryo transfer. Boxes are drawn from the first quartile (25th percentile) to the third quartile (75th percentile). Black lines in boxes are medians. Whiskers represent 10–90 percentiles. *N* is the number of patients. *p*-values are calculated by Mann–Whitney test. Comparison of diplotypes G C del/G C del, G C del/G C ins, and G C ins/G C ins by Kruskal–Wallis test, *p* = 0.0079.

We also found that patients exhibited different profiles of sHLA-G secretion according to their diplotypes. All statistical analysis concerning this part of the study is shown in Figure 5, Supplementary Tables 5–8. The most significant difference in concentration of sHLA-G measured in plasma before embryo transfer was achieved in patients with A-C-ins/G-C-del and G-C-ins/G-C-ins diplotypes (*p* = 0.007; median, 81.2 vs. 47.5 IU/ml, respectively). Patients with A-C-del/A-C-del diplotypes differed in concentration of sHLA-G with those with G-C-del/G-C-ins (*p* = 0.035; median, 112.1 vs. 28.9 IU/ml) and G-C-ins/G-C-ins (*p* = 0.022; median, 112.1 vs. 47.5 IU/ml). Moreover, G-C-del/G-C-del patients secreted a higher sHLA-G level than G-C-del/G-C-ins positive women (*p* = 0.019; median, 112.9 vs. 28.9 IU/ml), and G-C-ins/G-C-ins positive patients (*p* = 0.012; median, 112.9 vs. 47.5 IU/ml). In addition, G-G-del/G-C-del patients secreted a significantly higher sHLA-G level than G-C-del/G-C-ins patients (*p* = 0.048; median, 301.8 vs. 28.9 IU/ml), and G-C-ins/G-C-ins women (*p* = 0.036; median, 301.8 vs. 47.5 IU/ml). Higher quantities of sHLA-G were detected also for A-C-ins/G-C-del diplotypes than in G-C-del/G-C-ins positive women (*p* = 0.014; median, 81.2 vs. 28.9 IU/ml). Only in the case of G-C-del/G-C-ins diplotype did we observe a difference in the quantity of sHLA-G measured before and after IVF-ET (*p* = 0.044; median, 28.9 vs. 113.9 IU/ml). In addition, A-C-ins/A-C-del positive women secreted a higher median concentration of sHLA-G measured after embryo transfer than G-C-ins/G-C-ins women (*p* = 0.042; median, 100.2 vs. 56.3 IU/ml; Figure 5, Supplementary Table 5). Furthermore, when we compared secretion of sHLA-G in patients with rs1632947 G allele, who differed only in insertion allele (G-C-del/G-C-del vs. G-C-del/G-C-ins vs. G-C-ins/G-C-ins), we found significant differences among these diplotypes (*p* = 0.0079, Kruskal–Wallis test), while in comparisons of diplotypes A-C-del/A-C-del vs. A-C-ins/A-C-del vs. A-C-ins/A-C-ins, such differences were not observed (*p* = 0.43, Figure 5).

Summarizing the impact of *HLA-G* haplotypes and diplotypes on sHLA-G plasma level and pregnancy outcome in patients undergoing IVF-ET, we can conclude that polymorphisms in the *HLA-G* promoter region and 3′UTR influence expression and secretion of its soluble protein. Particular HLA-G haplotypes and diplotypes are associated with pregnancy outcome. Women positive for G-C-ins haplotype and G-C-ins/G-C-ins diplotype have the worst prognosis for pregnancy.

### sHLA-G Plasma Level of Patients Undergoing IVF-ET in Fresh or Frozen/Thawed Cycles

**Figure 6:**
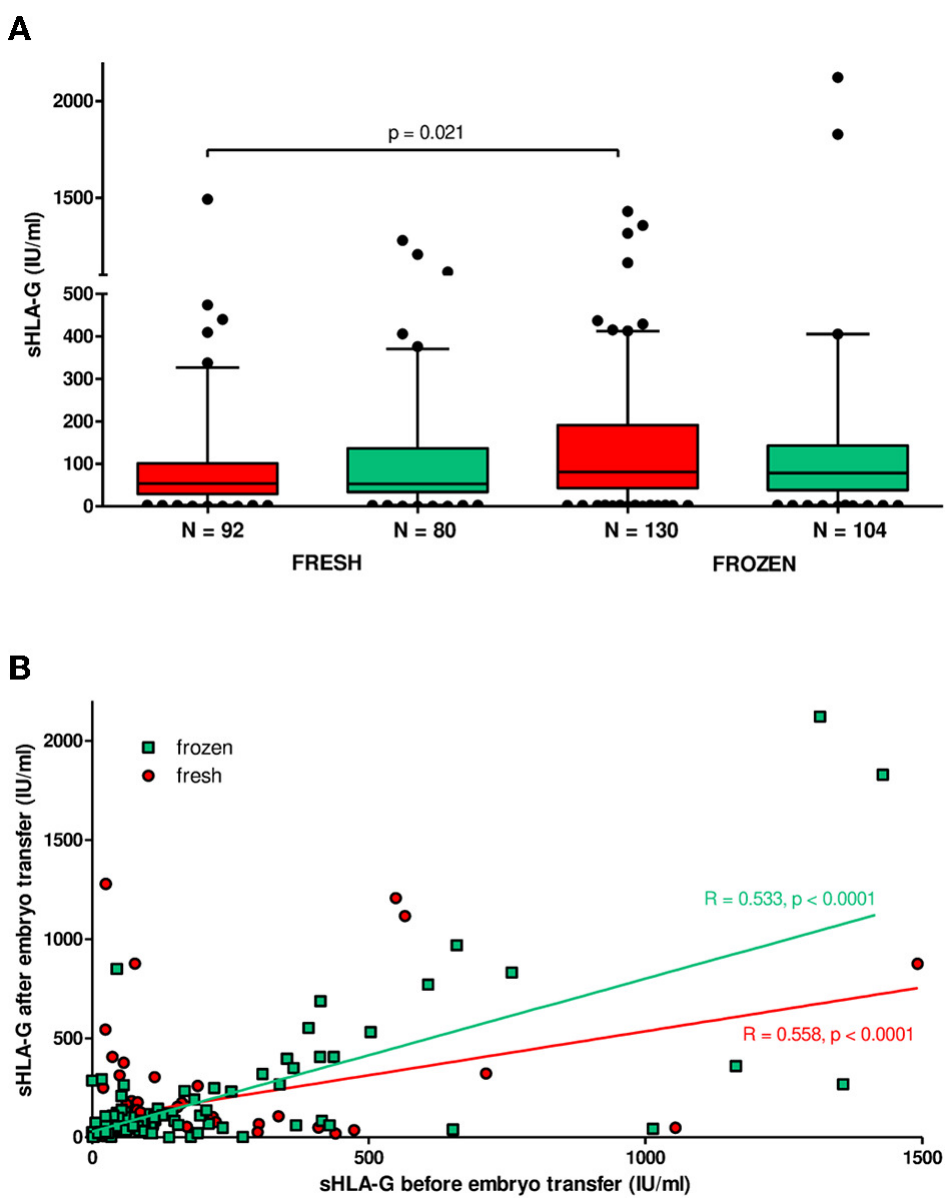
Soluble HLA-G plasma level in patients undergoing fresh or frozen/thawed cycles. **(A)** Median concentration of sHLA-G (black lines in boxes). Boxes are drawn from the first quartile (25th percentile) to the third quartile (75th percentile). Whiskers represent 10–90 percentiles. *N* is the number of patients. sHLA-G was measured in plasma of patients before (red boxes) and after embryo transfer (green boxes). *p*-value is calculated by Mann–Whitney test. **(B)** Correlation analysis obtained with Spearman correlation test. On the *x* and *y* axes are values for sHLA-G (IU/ml) before and after embryo transfer. Frozen cycle is represented by green line and fresh by red line (*R*, Spearman correlation coefficient; *p, p*-value).

**Figure 7:**
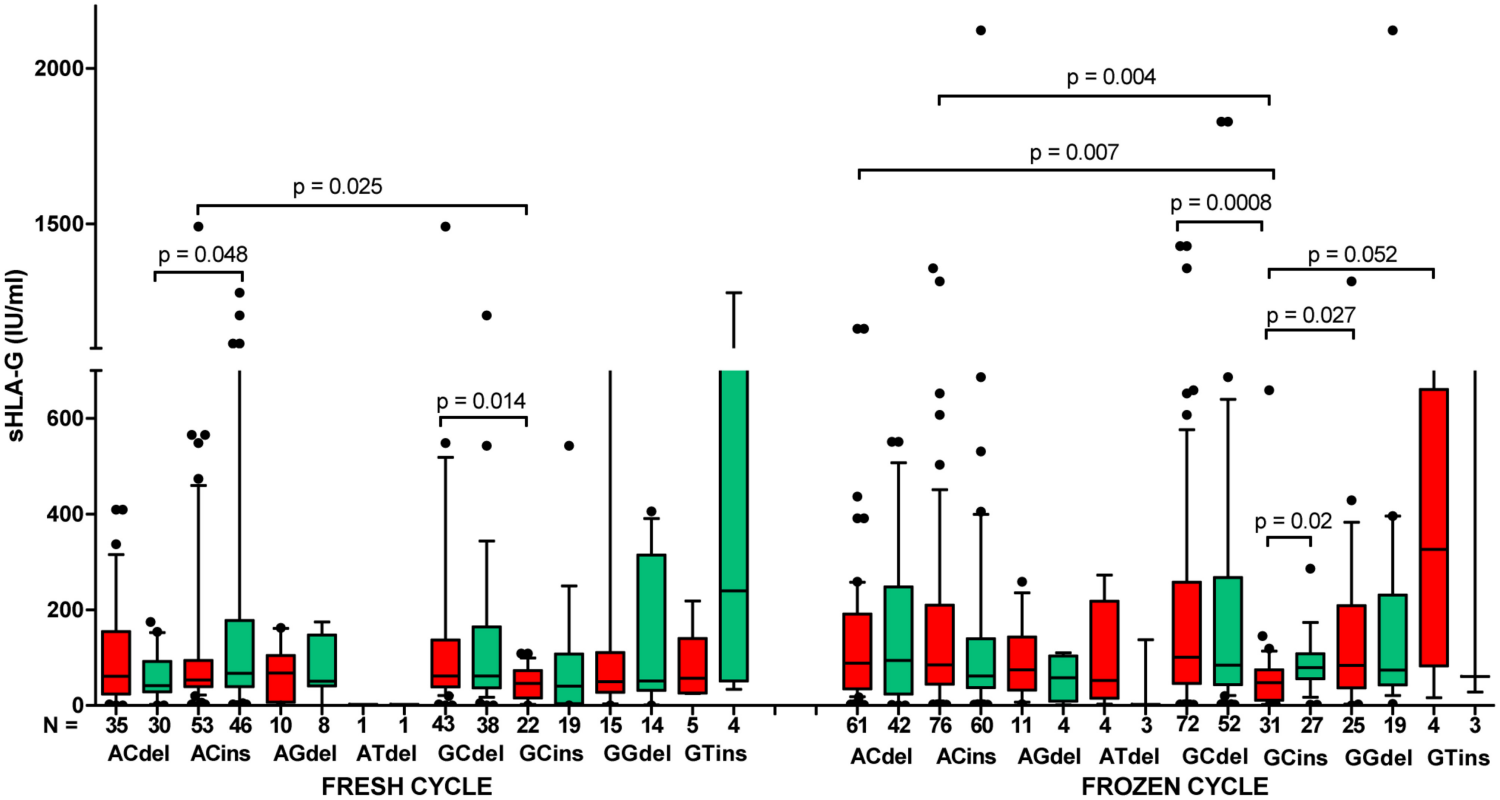
Concentration of soluble HLA-G per milliliter of plasma (IU/ml) in all patients according to their *HLA-G* haplotypes, dependent on fresh or frozen/thawed cycle. Haplotypes were estimated in the following order: rs1632947:−964G>A; rs1233334:−725G>C/T; rs371194629:insATTTGTTCATGCCT/del. Boxes are drawn from the first quartile (25th percentile) to the third quartile (75th percentile). Black lines in boxes are medians. Whiskers represent 10–90 percentiles. *N* is the number of patients. sHLA-G was measured in plasma of patients before (red boxes) and after embryo transfer (green boxes). *p*-values are calculated by Mann–Whitney test. Comparison of haplotypes G C del and G C ins (fresh vs. frozen cycle) by Kruskal–Wallis test, *p* = 0.0001.

Patients in frozen/thawed cycles secreted a significantly higher concentration of sHLA-G than patients in fresh cycles (*p* = 0.021, Figure 6A). Spearman correlation test showed positive correlation of sHLA-G measured before and after embryo transfer in frozen as well as fresh cycles; however, the frozen cycle (green line) seemed more beneficial for pregnancy than fresh cycle (red line), despite the similar Spearman coefficients *R* (*R* = 0.553, *p* < 0.0001 and *R* = 0.558, *p* < 0.0001, respectively; Figure 6B). This means that more frozen cycle patients achieved a higher level of sHLA-G after embryo transfer than fresh cycle patients. Moreover, the most significant difference in median concentration of sHLA-G measured before embryo transfer was observed between haplotypes in patients who underwent a frozen cycle. Namely, comparisons of G-C-del vs. G-C-ins (*p* = 0.0008; median, 100.9 vs. 47.58 IU/ml), A-C-del, A-C-ins vs. G-C-ins patients (*p* = 0.007, median, 88.64 vs. 47.58 IU/ml; *p* = 0.004, median, 84.61 vs. 47.58 IU/ml, respectively; Figure 7, Supplementary Table 9) were significant. Similar observations were detected in patients in fresh cycles, but to a lesser extent significant, perhaps due to lower number of patients with fresh cycles than with frozen cycles. In addition, comparison of sHLA-G secretion in patients who differed only in the insertion allele in G-C-del and G-C-ins haplotypes showed difference between patients of fresh and frozen cycles (*p* = 0.0001, Kruskal–Wallis test (Figure 7, Supplementary Table 9).

### sHLA-G Plasma Level in Different Ovarian Stimulation Protocols

**Figure 8:**
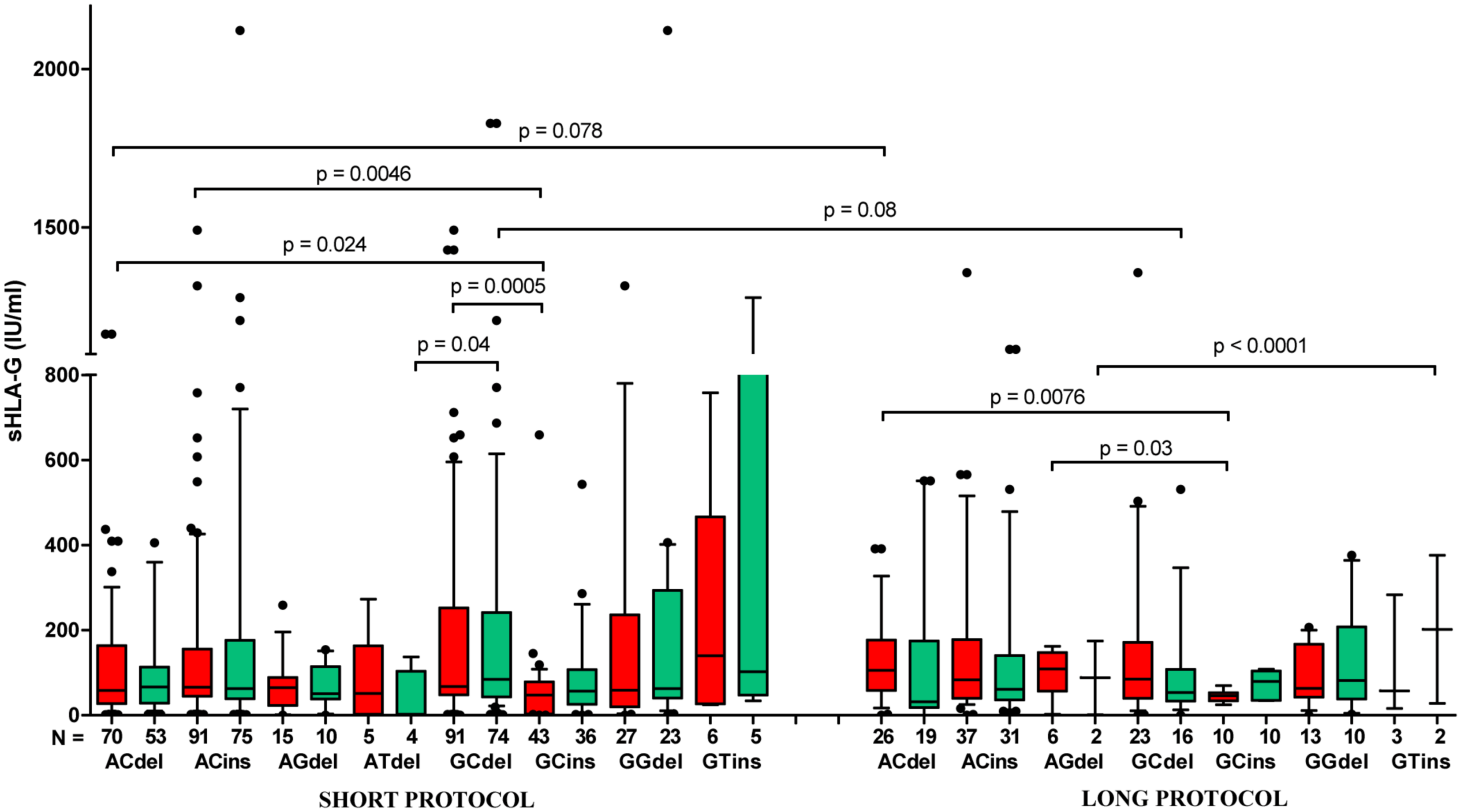
Concentration of soluble HLA-G per milliliter of plasma (IU/ml) in all patients according to their *HLA-G* haplotypes, dependent on short (with GnRH antagonist) or long (with GnRH agonist) ovarian stimulation protocol. Haplotypes were estimated in the following order: rs1632947:−964G>A; rs1233334:−725G>C/T; rs371194629:insATTTGTTCATGCCT/del. Boxes are drawn from the first quartile (25th percentile) to the third quartile (75th percentile). Black lines in boxes are medians. Whiskers represent 10–90 percentiles. *N* is the number of patients. sHLA-G was measured in plasma of patients before (red boxes) and after embryo transfer (green boxes). *p*-values are calculated by Mann–Whitney test. Comparison of haplotypes G C del and G C ins (short vs. long protocol) by Kruskal–Wallis test, *p* = 0.001.

When we compared the secretion of sHLA-G in women who underwent various ovarian stimulation protocols (short or long with GnRH antagonist or agonist, respectively), we did not observe a difference between them (Supplementary Figure 1A). Spearman correlation test showed positive correlation of sHLA-G measured before and after embryo transfer in short, as well as long, protocol; however, the long protocol (green line) seemed unfavorable to the success of the pregnancy than the short protocol (red line), and they differed in Spearman coefficients *R* (*R* = 0.607, *p* < 0.0001 and *R* = 0.499, *p* < 0.0001, respectively; Supplementary Figure 1B). Moreover, we found differences in sHLA-G level of patients with short protocol and positive for G-C-del, A-C-del, A-C-ins, and G-C-ins haplotypes (*p* = 0.0005, median, 67.66 vs. 47.58 IU/ml; *p* = 0.024, median, 58.25 vs. 47.58 IU/ml, *p* = 0.0046, median, 65.72 vs. 47.58 IU/ml, respectively). For long protocol, A-C-del haplotype positive women exhibited a higher sHLA-G level than G-C-ins women (*p* = 0.0076; median, 105.7 vs. 45.94 IU/ml, Figure 8, Supplementary Table 11). In addition, Kruskal–Wallis test indicated significant differences in sHLA-G concentration between patients undergoing short or long protocol, who were positive for G-C-del or G-C-ins haplotype (*p* = 0.001, Figure 8, Supplementary Table 11).

## Discussion

In this study, we investigated whether selected *HLA-G* polymorphisms and their protein levels measured in the plasma of patients undergoing IVF-ET could predispose an individual to infertility and RIF and influence pregnancy outcome. Owing to the fact that maternal and embryo immunoregulation may differ in spontaneous and IVF pregnancies, we compared the tested polymorphic variants between (i) all IVF patients with fertile controls, (ii) RIF patients with fertile controls, (iii) SIVF with fertile controls, and (iv) RIF with SIVF patients. Since the definition of RIF is not precisely defined and scientific literature indicates high heterogeneity of this disease (4), we have adopted RIF as at least three unsuccessful transfers of a good quality embryo to the uterus resulting in a lack of clinical pregnancy or its complication, namely, abortion. However, in our RIF patient group, there were, on average, four unsuccessful embryo transfers. Moreover, RIF and SIVF groups differed significantly in clinical manifestations and data, which is logical from a biological/medical point of view. It was to be expected that women who, after repeated embryo transfers, cannot conceive or maintain pregnancy will be different from those who become pregnant. It is worth emphasizing that, in our whole patient group, ~30% of cases had unknown causes of infertility. According to scientific sources, unexplained infertility affects 10–50% of couples seeking infertility care (47–49).

We have deduced from our research that searching for single loci in *HLA-G* gene as a risk marker for infertility did not give the results expected. Only GG genotype in promoter rs1632947: c.-964G>A was linked with protection against infertility, while the most studied *HLA-G* polymorphism in the field of reproduction, rs371194629: c.^*^65_^*^66insATTTGTTCATGCCT in 3′UTR of exon 8, had a similar distribution in our group of patients and controls (Table 2). Other epidemiological studies concerning the role of 14-bp ins/del polymorphism in RIF provided apparently inconclusive results, which might be due to differences in the studied populations and limited sample sizes (50–52). However, a recently published meta-analysis (37) on the impact of 14-bp ins/del polymorphism in recurrent miscarriage after natural or artificial conception revealed that women of European countries with the HLA-G 14-bp insertion/insertion homozygous genotype have a significantly higher prevalence of recurrent miscarriage. A study among another Polish group (53) indicated that the frequencies of the 14-bp ins/del genotypes in the 3′-UTR of the *HLA-G* gene in groups with reproductive disorders did not report significant differences with the control group, and this result is concordant with our study. However, they suggest that the risk of complications in pregnancy is influenced rather by the particular alleles like HLA-G 10101, HLA-G 10108, and HLA-G 10106, not SNPs. Therefore, in our study, the analysis of haplotypes and diplotypes was substantively justified. We found in relatively high numbers of patients and control women that certain *HLA-G* haplotypes and diplotypes were associated in general with infertility, while others were protective (Tables 3, 4). We consider this because differences were also found between successful IVF patients and women conceiving naturally. We consider disease-associated haplotypes that were determined by genetic estimation, A-C-del, G-C-ins, and A-G-del, should be linked with lower secretion of sHLA-G, whereas protective, A-C-ins, G-C-del, and G-G-del with higher secretion, as lower levels of sHLA-G have been reported to be associated with pregnancy failures (20, 21). In the present study, the lowest secretors were G-C-ins haplotype carriers, while the highest were G-C-del carriers; other carriers were intermediate secretors (Figure 2, red boxes; Supplementary Table 1). Interestingly, when we looked at diplotypes, a 14-bp insertion allele-dependent risk was observed (Table 4). ORs of infertility appearance increased from 0.38 for G-C-del/G-C-del women to 18.20 for G-C-del/G-C-ins and even to 48.63 for G-C-ins/G-C-ins. This last diplotype is undoubtedly very disadvantageous for women who want to conceive naturally. Not a single woman from our group of 320 fertile women possessed this diplotype (Table 4). The measurement of sHLA-G in the plasma of patients before embryo transfer confirmed our genetic study. G-C-del carriers secreted significantly higher levels of sHLA-G in comparison to G-C-ins carriers, which was shown in haplotype as well as diplotype comparisons (Figures 2, 5). From the haplotype analysis, A-G-del haplotype seemed to predispose to infertility, but statistical analysis proved to be uninformative due to the wide confidence interval (Table 3). In fact, this ambiguity was seen then in diplotype frequencies where the A-C-del/A-G-del diplotype had a predisposing effect, but A-G-del/A-G-del diplotype was protective (Table 4). Indeed, when we looked at the secretion of sHLA-G in patients positive for these diplotypes before embryo transfer, we observed that A-C-del/A-G-del patients had 5.618 IU/ml median concentration, whereas A-G-del/A-G-del positive patients had 108.7 IU/ml (Supplementary Table 7). Another point which should be emphasized here is that not always is the 14-bp insertion allele associated with susceptibility to pregnancy failure and the deletion allele with successful pregnancy. In our study, 14-bp insertion allele in combination with A allele in position −964G>A and C allele in position −725G>C/T was more prevalent in fertile controls than in IVF patients. In contrast, 14-bp deletion allele in the above mentioned combination was in higher frequency in IVF patients than in fertile controls (Table 3). However, secretion of sHLA-G from A-C-ins and A-C-del haplotype IVF patients measured before embryo transfer was similar (Supplementary Table 1). Maybe, this explains why some studies have shown discrepant results concerning the role of 14-bp ins/del polymorphism in reproductive failures. Moreover, when we divided IVF patients according to only 14-bp ins/del polymorphism and looked at their secretion of sHLA-G before an embryo was transferred, we found higher secretion in del/del patients in comparison to ins/ins patients, but this difference was not high (*p* = 0.045, data not shown). This suggests again that the studies should not correlate sHLA-G measurement to one polymorphism, e.g., 14-bp ins/del, because the level of HLA-G protein secreted to plasma depends on a genetic combination of many SNPs, especially in the promoter region, which are close to regulatory elements and CpG sites, and could alter binding of transcription factors or promoter methylation and therefore impact the rate of transcription (12). Moreover, Castelli et al. (38) created extended haplotypes consisting of promoter and 3′UTR haplotypes, indicating G010101a/G^*^01:01:01:01/UTR-1 (in our study G-C-del haplotype, respectively) as the most frequent haplotype worldwide (38). This haplotype is evolutionarily favored and must be associated with human survival; therefore, it makes sense that it is more common in our group of fertile women. Moreover, our diplotype frequency analysis (Table 4) and other studies showed evidence concerning balancing selection acting on the *HLA-G* promoter (40, 54), suggesting that promoters have been maintained with high heterozygosity. In our study, heterozygotic diplotypes make up ~70% of all diplotypes. It is probably related to a possible better adaptation of individuals carrying both high- and low-expressing promoters. Therefore, divergent *HLA-G* promoter haplotypes/diplotypes are associated with differential HLA-G expression as was noticed by Castelli et al. (39) and Rebmann et al. (55) who reported G^*^01013 (+14 bp) and G^*^01015 N (+14 bp) alleles as “low secretor” alleles, while G^*^01041 (−14 bp) as the “high secretor” allele (39, 55).

Costa et al. presented the HLA-G profile of couples with an unknown cause of infertility and who underwent ART (56). According to their study *HLA-G* promoter haplotype 010101b was considered as a susceptibility allele for infertility. This haplotype possesses G allele in position −964G>A and also G allele in −725G>C/T. It is in opposition to our results because haplotypes containing G allele in both tested SNPs were protective in our analysis. Moreover, G allele in −725G>C/T position was previously described to be correlated with higher *HLA-G* expression levels (42). Since higher sHLA-G secretion is associated with reproductive success, and the G allele at −725G>C/T position increases gene expression, we believe our research is close to the truth, at least for the European population. In addition, another argument in favor of our research was a positive correlation of plasma sHLA-G for G-G-del haplotypes with pregnancy (Figure 4F).

Unfortunately, we had no access to plasma of women who naturally conceived; therefore, we could not compare sHLA-G level in IVF patients with fertile women according to their haplotypes/diplotypes. This is a limitation of our study. In the case of fertile groups, it would be very difficult to collect a sufficient number of plasma samples before women get naturally pregnant and again in early pregnancy (~2 weeks after fertilization) as we did for IVF patients. However, in this study for the first time, the level of sHLA-G was measured before and after embryo transfer to check the impact of its concentration on pregnancy outcome as early as possible and also its haplotype/diplotype dependent secretion. Generally, women who become pregnant after IVF-ET expressed higher sHLA-G measured after embryo transfer. When embryo transfer was unsuccessful, we saw that there was a decrease in sHLA-G level. Moreover, G-C-del women secreted about two times more sHLA-G than G-C-ins women, which was observed in IVF transfers with pregnancy (Table 5, Supplementary Table 2). The difference in secretion between these haplotype carriers was widened when embryo transfer ended in miscarriage (Figure 3C). Marozio et al. reported sHLA-G levels lower than 43.50 IU/ml at the end of the first trimester to be associated with a two-fold increased risk of developing a pregnancy complication (57). In our study, 59.73 IU/ml sHLA-G was threshold value above which patient was with almost two-fold increased chance to get pregnant than patient who secreted below this value. In another study performed by Pfeiffer et al. (21), it was revealed that low sHLA-G levels measured in preovulatory women appear to be at risk for early abortion after IVF (21). Therefore, genetic haplotype/diplotype determination could be useful in prediction of infertility risk before IVF-ET. In our study, correlation analysis of soluble HLA-G level measured before and after embryo transfer for particular women with their pregnancy outcome showed a similar pattern for A-C-ins and G-C-del haplotype positive women (Figures 4B,D), which speaks indeed for the protective effect of the A-C-ins haplotype disclosed genetically. Once again, G-C-ins haplotype (Figure 4E) and G-C-ins/G-C-ins diplotype (Figure 5) seems the most disadvantageous for achieving pregnancy.

In addition, we found that IVF patients in frozen/thawed cycles secreted higher sHLA-G than patients in fresh cycles. It means that patients from frozen cycles would be at a relatively lower risk of pregnancy complications. Actually, recent worldwide data point to improving live birth rates in frozen–thawed cycles in all regions (58). Subsequently, Maheshwari et al. (59) reported singleton babies conceived from frozen/thawed embryos who were at a lower relative risk of preterm delivery, low birth weight, and small for gestational age compared to those conceived from fresh embryo transfers (59). The explanation for this phenomenon may be the fact that women in frozen cycles have time to reach a physiological hormonal state that may affect HLA-G expression. Progesterone is an immunomodulatory steroid hormone secreted by the corpus luteum and placenta, allowing endometrium maintenance and embryo implantation. Progesterone could induce HLA-G expression by progesterone response element, which is located in the *HLA-G* promoter between positions −52 and −38 of the gene sequence (11, 60). Moreover, exogenous administration of progesterone to patients may increase HLA-G expression and have a beneficial effect on pregnancy. Cochrane Systematic Review as well as Practice Committee of American Society for Reproductive Medicine confirms the essential role of progesterone supplementation in luteal phase support in patients undergoing ART procedures (61–63). It worth to mention the recent work performed by Nguyen et al. (64) who demonstrated an influence of hormones on the HLA-G secretion in congenital adrenal hyperplasia patients. These patients secreted higher HLA-G levels than healthy controls. Moreover, HLA-G level was positively associated with progesterone and corticosteroid supplementation and negatively with estradiol. This may also suggest a role of the renin–angiotensin system in the expression of soluble HLA-G (64).

The last analysis to be commented on is the dependence of sHLA-G secretion on the applied ovarian stimulation protocol. Toftager et al. (65) observed that the chances of at least one live birth after use of fresh and frozen embryos after the first ART cycle are similar in GnRH antagonist (short) as well as GnRH agonist (long) protocols (65). In addition, Tomás et al. found similar perinatal outcomes after the GnRH antagonist vs. GnRH agonist ovarian stimulation protocols in fresh and frozen cycles (66). However, these studies did not analyze HLA-G secretion and *HLA-G* polymorphism. Moreover, in the Tomás et al. study, embryo transfers ended in pregnancy for all patients who were qualified for the analyses (66). In our work, the analysis concerned patients, regardless of whether it was successful or not. Maybe, if they took all the patients who became pregnant and those who did not, these differences would be visible. There is no literature data correlating in one study *HLA-G* polymorphism with the level of secreted sHLA-G with the kind of procedure (fresh or frozen cycle), as well as with the type of ovarian stimulation protocol and with pregnancy outcome. Our study did not find differences in secretion of sHLA-G in all patients undergoing short or long protocols (Supplementary Figure 1A). However, correlation analysis indicated short protocol as more beneficial for the outcome of pregnancy (Supplementary Figure 1B). Concentration of soluble HLA-G was also haplotype dependent, although patients were administered to use exogenous progesterone, suggesting that endogenous secretion of sHLA-G is important not only for the pregnancy outcome but also in immunoregulatory function required to proper development and function of our organism.

We also feel that a defect or deletion of Enhancer L may be a cause of the absence of HLA-G expression and secretion of sHLA-G to plasma patients (67). Indeed, we observed that, in some patients' plasma (even with the most advantageous G-C-del haplotype), we could not detect sHLA-G. It seems that it would be worth investigating the role of Enhancer L in these patients. This could be the future direction of next research.

## Conclusions

We can conclude the following statements:

- Infertility is associated with *HLA-G* polymorphism.
- Polymorphisms in the *HLA-G* promoter region and 3′UTR influence expression and secretion of its soluble protein.
- Particular *HLA-G* haplotypes and diplotypes were associated with pregnancy outcome.
- Regardless of possessed haplotype by the patient, 59.73 IU/ml sHLA-G was the threshold value above which patient was with almost two-fold increased chance to get pregnant than patient who secreted below this value.
- IVF patients in frozen/thawed cycles secreted higher sHLA-G than patients in fresh cycles.
- Short ovarian stimulation protocol with GnRH antagonist seemed more beneficial than long protocol with GnRH agonist.

## Data Availability Statement

All relevant data is contained within the article. All datasets analyzed for this study are included in the article/Supplementary Files.

## Ethics Statement

The studies involving human participants were reviewed and approved by the Medical University of Wrocław and Polish Mothers' Memorial Hospital-Research Institute in Łódź. The patients/participants provided their written informed consent to participate in this study.

## Author Contributions

IN conceived and designed the experiments. IN, KW, and AW performed the experiments and analyzed the data. PR, MR, JW, AM, and RK contributed to patients and control recruitments. IN and KW wrote the paper. PK critically reviewed the paper.

### Conflict of Interest

The authors declare that the research was conducted in the absence of any commercial or financial relationships that could be construed as a potential conflict of interest.
