# Supplementary material for: Association of Soluble HLA-G Plasma Level and HLA-G Genetic Polymorphism With Pregnancy Outcome of Patients Undergoing in vitro Fertilization Embryo Transfer
Source: Front Immunol. 2020 Jan 14;10:2982. doi: 10.3389/fimmu.2019.02982 (PMC6971053; doi:10.3389/fimmu.2019.02982)
Supplement: Supplementary file 1 [file Table_1.DOCX]

**Supplementary Table 1** HLA-G value (IU/ml) measured before and after IVF embryo transfer in all patients depending on particular *HLA-G* haplotypes

*Haplotypes were estimated in the following order: rs1632947:-964G>A; rs1233334:-725G>C/T; rs371194629:insATTTGTTCATGCCT/del. P values are calculated by Mann-Whitney test.

| **Haplotype*** | **A C del** | | **A C ins** | | **A G del** | | **A T del** | | **G C del** | | **G C ins** | | **G G del** | | **G T ins** | |
| --- | --- | --- | --- | --- | --- | --- | --- | --- | --- | --- | --- | --- | --- | --- | --- | --- |
| **Before or after IVF-ET** | **before** | **after** | **before** | **after** | **before** | **after** | **before** | **after** | **before** | **after** | **before** | **after** | **before** | **after** | **before** | **after** |
| Number of patients | 96 | 72 | 129 | 106 | 21 | 12 | 5 | 4 | 115 | 90 | 53 | 46 | 40 | 33 | 9 | 7 |
| Minimum | 0.0 | 0.0 | 0.0 | 0.0 | 0.0 | 0.0 | 2.256 | 2.037 | 0.0 | 0.0 | 0.0 | 0.0 | 0.0 | 0.0 | 16.23 | 27.88 |
| 25% Percentile | 30.58 | 25.14 | 42.36 | 38.55 | 27.69 | 33.70 | 2.591 | 2.073 | 45.20 | 42.11 | 11.18 | 34.91 | 33.05 | 41.30 | 26.28 | 33.92 |
| Median | 69.02 | **57.36^a^** | 65.72 | 62.53 | 70.66 | 50.78 | 51.39 | 2.363 | 79.95 | 75.43 | **47.34^b, c, d, e, f, g^** | 59.44 | 61.50 | 69.29 | 61.49 | 102.6 |
| 75% Percentile | 160.2 | 123.1 | 161.4 | 146.0 | 135.1 | 122.3 | 163.2 | 103.6 | 252.3 | 189.8 | 73.59 | 107.5 | 189.4 | 261.3 | 326.0 | 831.1 |
| Maximum | 1163 | 876.9 | 1492 | 2122 | 258.5 | 174.8 | 272.7 | 137.3 | 1492 | 1828 | 658.8 | 968.9 | 1315 | 2122 | 758.1 | 1278 |
| Mean | 131.4 | 129.9 | 163.9 | 185.6 | 80.05 | 72.23 | 76.61 | 36.00 | 202.0 | 197.8 | 57.61 | 102.7 | 166.0 | 190.1 | 201.8 | 387.2 |
| Std. Deviation | 185.6 | 193.2 | 256.9 | 330.5 | 65.69 | 57.58 | 112.4 | 67.50 | 305.6 | 330.6 | 91.53 | 160.6 | 275.2 | 369.3 | 245.2 | 488.7 |
| Std. Error | 18.94 | 22.77 | 22.61 | 32.10 | 14.34 | 16.62 | 50.28 | 33.75 | 28.49 | 34.85 | 12.57 | 23.68 | 43.51 | 64.29 | 81.73 | 184.7 |
| Lower 95% CI of mean | 93.79 | 84.50 | 119.1 | 122.0 | 50.14 | 35.65 | -63.00 | -71.40 | 145.6 | 128.6 | 32.38 | 55.05 | 77.95 | 59.19 | 13.31 | -64.76 |
| Upper 95% CI of mean | 169.0 | 175.3 | 208.6 | 249.3 | 109.9 | 108.8 | 216.2 | 143.4 | 258.5 | 267.0 | 82.84 | 150.4 | 254.0 | 321.1 | 390.2 | 839.3 |
| D'Agostino & Pearson omnibus normality test K^2^ | 107.9 | 57.03 | 110.4 | 99.48 | 5.305 | 1.215 | N too small | N too small | 84.61 | 89.62 | 99.95 | 70.95 | 46.53 | 67.47 | 8.385 | N too small |

^a^ A C del after vs G C del after: p = 0.058; ^b^ A C del before vs G C ins before: p = 0.0009; ^c^ A C ins before vs G C ins before: p = 0.0003; ^d^ A G del before vs G C ins before: p = 0.057;

^e^ G C del before vs G C ins before: p < 0.0001; ^f^ G G del before vs G C ins before: p = 0.017; ^g^ G C ins before vs after: p = 0.032
