# Supplementary material for: Association of Soluble HLA-G Plasma Level and HLA-G Genetic Polymorphism With Pregnancy Outcome of Patients Undergoing in vitro Fertilization Embryo Transfer
Source: Front Immunol. 2020 Jan 14;10:2982. doi: 10.3389/fimmu.2019.02982 (PMC6971053; doi:10.3389/fimmu.2019.02982)
Supplement: Supplementary file 2 [file Table_2.DOCX]

**Supplementary Table 2** HLA-G value (IU/ml) measured before and after IVF embryo transfer in patients who achieved clinical pregnancy, depending on particular *HLA-G* haplotypes

*Haplotypes were estimated in the following order: rs1632947:-964G>A; rs1233334:-725G>C/T; rs371194629:insATTTGTTCATGCCT/del

| **Haplotype*** | **A C del** | | **A C ins** | | **A G del** | | **A T del** | | **G C del** | | **G C ins** | | **G G del** | | **G T ins** | |
| --- | --- | --- | --- | --- | --- | --- | --- | --- | --- | --- | --- | --- | --- | --- | --- | --- |
| **Before or after IVF-ET** | **before** | **after** | **before** | **after** | **before** | **after** | **before** | **after** | **before** | **after** | **before** | **after** | **before** | **after** | **before** | **after** |
| Number of patients | 49 | 45 | 75 | 65 | 9 | 8 | 3 | 3 | 67 | 60 | 28 | 26 | 27 | 22 | 6 | 5 |
| Minimum | 0.0 | 0.0 | 0.0 | 0.0 | 2.204 | 40.72 | 2.925 | 2.037 | 0.0 | 0.0 | 0.0 | 0.0 | 0.0 | 0.0 | 24.90 | 33.92 |
| 25% Percentile | 26.46 | 28.35 | 45.44 | 35.53 | 67.91 | 50.78 | 2.925 | 2.037 | 43.24 | 37.63 | 11.18 | 35.01 | 17.38 | 25.57 | 26.96 | 47.27 |
| Median | 58.95 | 66.78 | 74.56 | 66.21 | 87.74 | 97.22 | 53.77 | 2.182 | 79.95 | 71.17 | **37.21^a, b, c, d, e, f^** | 73.47 | 63.38 | 68.44 | 139.9 | 102.6 |
| 75% Percentile | 120.2 | 113.1 | 184.7 | 199.8 | 140.8 | 147.2 | 272.7 | 137.3 | 251.4 | 167.2 | 82.72 | 111.4 | 149.3 | 200.8 | 466.1 | 1055 |
| Maximum | 391.5 | 876.9 | 1492 | 2122 | 162.2 | 174.8 | 272.7 | 137.3 | 1492 | 1828 | 145.1 | 543.0 | 1315 | 2122 | 758.1 | 1278 |
| Mean | 95.99 | 145.9 | 191.7 | 242.9 | 92.09 | 99.01 | 109.8 | 47.16 | 212.7 | 215.0 | 47.67 | 105.9 | 155.1 | 194.7 | 243.2 | 461.4 |
| Std. Deviation | 100.7 | 224.1 | 286.9 | 403.7 | 49.71 | 50.60 | 143.3 | 78.02 | 333.1 | 378.8 | 42.44 | 116.6 | 278.1 | 442.4 | 285.9 | 564.9 |
| Std. Error | 14.39 | 33.41 | 33.13 | 50.07 | 16.57 | 17.89 | 82.76 | 45.05 | 40.70 | 48.91 | 8.021 | 22.86 | 53.52 | 94.32 | 116.7 | 252.6 |
| Lower 95% CI of mean | 67.05 | 78.55 | 125.7 | 142.8 | 53.87 | 56.71 | -246.3 | -146.7 | 131.5 | 117.1 | 31.21 | 58.80 | 45.13 | -1.452 | -56.81 | -240.1 |
| Upper 95% CI of mean | 124.9 | 213.2 | 257.7 | 342.9 | 130.3 | 141.3 | 465.9 | 241.0 | 294.0 | 312.9 | 64.12 | 153.0 | 265.1 | 390.8 | 543.2 | 1163 |
| D'Agostino & Pearson omnibus normality test K^2^ | 21.06 | 37.34 | 61.07 | 52.76 | 0.1406 | 1.411 | N too small | N too small | 54.72 | 62.04 | 3.153 | 29.40 | 42.83 | 51.71 | N too small | N too small |

^a^ A C del before vs G C ins before: p = 0.04; ^b^ A C ins before vs G C ins before: p = 0.016; ^c^ A G del before vs G C ins before: p = 0.046; ^d^ G C del before vs G C ins before: p = 0.014;

^e^ G T ins before vs G C ins before: p = 0.049; ^f^ G C ins before vs after: p = 0.033
