# Supplementary material for: Association of Soluble HLA-G Plasma Level and HLA-G Genetic Polymorphism With Pregnancy Outcome of Patients Undergoing in vitro Fertilization Embryo Transfer
Source: Front Immunol. 2020 Jan 14;10:2982. doi: 10.3389/fimmu.2019.02982 (PMC6971053; doi:10.3389/fimmu.2019.02982)
Supplement: Supplementary file 3 [file Table_3.DOCX]

**Supplementary Table 3** HLA-G value (IU/ml) measured before and after IVF embryo transfer in patients with a lack of pregnancy, depending on particular *HLA-G* haplotypes

*Haplotypes were estimated in the following order: rs1632947:-964G>A; rs1233334:-725G>C/T; rs371194629:insATTTGTTCATGCCT/del

^a^ A C del before vs A C ins before: p = 0.05

| **Haplotype*** | **A C del** | | **A C ins** | | **A G del** | | **A T del** | | **G C del** | | **G C ins** | | **G G del** | | **G T ins** | |
| --- | --- | --- | --- | --- | --- | --- | --- | --- | --- | --- | --- | --- | --- | --- | --- | --- |
| **Before or after IVF-ET** | **before** | **after** | **before** | **after** | **before** | **after** | **before** | **after** | **before** | **after** | **before** | **after** | **before** | **after** | **before** | **after** |
| Number of patients | 21 | 12 | 23 | 15 | 6 | 1 | 2 | 1 | 22 | 12 | 7 | 4 | 7 | 6 | 2 | 1 |
| Minimum | 2.668 | 1.529 | 2.109 | 2.544 | 2.668 | 1.529 | 2.256 | 2.544 | 2.109 | 2.715 | 21.91 | 39.88 | 39.64 | 42.39 | 57.19 | 376.1 |
| 25% Percentile | 59.38 | 48.78 | 42.36 | 39.02 | 7.092 | 1.529 | 2.256 | 2.544 | 48.87 | 42.93 | 52.29 | 39.88 | 48.87 | 43.00 | 57.19 | 376.1 |
| Median | **113.9^a^** | 98.25 | 53.59 | 42.84 | 74.45 | 1.529 | 26.82 | 2.544 | 73.49 | 56.04 | 61.20 | 46.32 | 92.16 | 58.48 | 170.3 | 376.1 |
| 75% Percentile | 208.0 | 220.6 | 113.9 | 87.08 | 142.9 | 1.529 | 51.39 | 2.544 | 297.3 | 267.6 | 78.60 | 739.9 | 211.0 | 288.4 | 283.4 | 376.1 |
| Maximum | 436.8 | 405.3 | 1357 | 268.1 | 142.9 | 1.529 | 51.39 | 2.544 | 1357 | 968.9 | 658.8 | 968.9 | 1054 | 376.1 | 283.4 | 376.1 |
| Mean | 153.2 | 128.7 | 142.6 | 85.67 | 74.32 | 1.529 | 26.82 | 2.544 | 240.4 | 194.1 | 144.7 | 275.4 | 242.0 | 139.6 | 170.3 | 376.1 |
| Std. Deviation | 127.3 | 116.4 | 278.3 | 94.83 | 61.42 | 0.00 | 34.74 | 0.0 | 355.0 | 274.1 | 227.5 | 462.4 | 364.7 | 143.1 | 159.9 | 0.0 |
| Std. Error | 27.79 | 33.61 | 58.03 | 24.49 | 25.07 | 0.00 | 24.57 | 0.0 | 75.68 | 79.12 | 86.00 | 231.2 | 137.9 | 58.41 | 113.1 | 0.0 |
| Lower 95% CI of mean | 95.25 | 54.77 | 22.27 | 33.16 | 9.864 | 0.00 | -285.3 | 0.00 | 83.01 | 19.98 | -65.77 | -460.4 | -95.33 | -10.53 | -1267 | 0.00 |
| Upper 95% CI of mean | 211.2 | 202.7 | 263.0 | 138.2 | 138.8 | 0.00 | 339.0 | 0.00 | 397.8 | 368.2 | 355.1 | 1011 | 579.3 | 289.8 | 1607 | 0.00 |
| D'Agostino & Pearson omnibus normality test K^2^ | 6.301 | 6.494 | 50.49 | 6.412 | N too small | N too small | N too small | N too small | 21.72 | 19.37 | N too small | N too small | N too small | N too small | N too small | N too small |
