# Supplementary material for: Association of Soluble HLA-G Plasma Level and HLA-G Genetic Polymorphism With Pregnancy Outcome of Patients Undergoing in vitro Fertilization Embryo Transfer
Source: Front Immunol. 2020 Jan 14;10:2982. doi: 10.3389/fimmu.2019.02982 (PMC6971053; doi:10.3389/fimmu.2019.02982)
Supplement: Supplementary file 4 [file Table_4.DOCX]

**Supplementary Table 4** HLA-G value (IU/ml) measured before and after IVF embryo transfer in patients with miscarriage, depending on particular *HLA-G* haplotypes

*Haplotypes were estimated in the following order: rs1632947:-964G>A; rs1233334:-725G>C/T; rs371194629:insATTTGTTCATGCCT/del

| **Haplotype*** | **A C del** | | **A C ins** | | **A G del** | | **G C del** | | **G C ins** | | **G G del** | | **G T ins** | |
| --- | --- | --- | --- | --- | --- | --- | --- | --- | --- | --- | --- | --- | --- | --- |
| **Before or after IVF-ET** | **before** | **after** | **before** | **after** | **before** | **after** | **before** | **after** | **before** | **after** | **before** | **after** | **before** | **after** |
| Number of patients | 26 | 15 | 31 | 26 | 6 | 3 | 26 | 18 | 18 | 16 | 6 | 5 | 1 | 1 |
| Minimum | 0.0 | 0.0 | 2.109 | 2.710 | 0.0 | 0.0 | 2.109 | 2.710 | 2.111 | 1.776 | 31.85 | 40.22 | 16.23 | 27.88 |
| 25% Percentile | 29.94 | 2.162 | 37.01 | 39.19 | 17.40 | 0.0^e^ | 47.32 | 43.18 | 2.461 | 11.60 | 35.45 | 44.25 | 16.23 | 27.88 |
| Median | **64.10^a^** | 25.01 | 71.83 | **67.46^b, c^** | 35.87 | 31.36 | 80.77 | **80.73^d, e, f^** | **46.76^g, h, i^** | 56.50 | 49.70 | 263.6 | 16.23 | 27.88 |
| 75% Percentile | 253.2 | 127.6 | 105.7 | 104.1 | 104.2 | 41.80 | 230.9 | 208.0 | 66.11 | 95.28 | 265.0 | 400.9 | 16.23 | 27.88 |
| Maximum | 1163 | 359.8 | 503.4 | 531.1 | 258.5 | 41.80 | 503.4 | 531.1 | 74.30 | 102.8 | 352.2 | 405.7 | 16.23 | 27.88 |
| Mean | 180.5 | 82.85 | 112.3 | 100.1 | 67.71 | 24.39 | 141.9 | 142.9 | 39.23 | 54.44 | 126.0 | 230.8 | 16.23 | 27.88 |
| Std. Deviation | 305.0 | 132.5 | 128.9 | 120.3 | 95.14 | 21.76 | 147.0 | 145.5 | 29.18 | 38.51 | 135.5 | 179.3 | 0.00 | 0.00 |
| Std. Error | 59.82 | 34.20 | 23.15 | 23.60 | 38.84 | 12.56 | 28.83 | 34.29 | 6.878 | 9.627 | 55.34 | 80.19 | 0.00 | 0.00 |
| Lower 95% CI of mean | 57.29 | 9.495 | 65.06 | 51.54 | -32.14 | -29.66 | 82.53 | 70.51 | 24.72 | 33.93 | -16.24 | 8.116 | 0.00 | 0.00 |
| Upper 95% CI of mean | 303.7 | 156.2 | 159.6 | 148.7 | 167.6 | 78.43 | 201.3 | 215.2 | 53.74 | 74.96 | 268.3 | 453.4 | 0.00 | 0.00 |
| D'Agostino & Pearson omnibus normality test K^2^ | 33.04 | 7.403 | 24.43 | 30.84 | N too small | N too small | 8.825 | 9.974 | 8.504 | 4.295 | N too small | N too small | N too small | N too small |

^a^ A C del before vs A C del after: p = 0.045; ^b^ A C del after vs A C ins after: p = 0.022; ^c^ A C ins after vs A G del after: p = 0.049; ^d^ A G del after vs G C del after: p = 0.031; ^e^ A C del after vs G C del after: p = 0.013; ^f^ A C del after vs GG del after: p = 0.023; ^g^ G C del before vs G C ins before: p = 0.0067; ^h^ A C ins before vs G C ins before: p = 0.013; ^i^ A C del before vs G C ins before: p = 0.052
