# Supplementary material for: Association of Soluble HLA-G Plasma Level and HLA-G Genetic Polymorphism With Pregnancy Outcome of Patients Undergoing in vitro Fertilization Embryo Transfer
Source: Front Immunol. 2020 Jan 14;10:2982. doi: 10.3389/fimmu.2019.02982 (PMC6971053; doi:10.3389/fimmu.2019.02982)
Supplement: Supplementary file 5 [file Table_5.DOCX]

**Supplementary Table 5** HLA-G value (IU/ml) measured before and after IVF embryo transfer in all patients according to particular *HLA-G* diplotypes

Diplotypes were determined from haplotype analysis and estimated in the following order: rs1632947:-964G>A; rs1233334:-725G>C/T; rs371194629:insATTTGTTCATGCCT/del. P values are calculated by Mann-Whitney test. ^a^ A C del/ A C del before vs G C del/ G C ins before: p = 0.035; ^b^ A C del/ A C del before vs G C ins/ G C ins before: p = 0.022; ^c^ A C ins/ A C del after vs G C ins/ G C ins after: p = 0.042; ^d^ A C ins/ G C del before vs G C del/ G C ins before: p = 0.014; ^e^ A C ins/ G C del before vs G C ins/ G C ins before: p = 0.007; ^f^ G C del/ G C del before vs G C del/ G C ins before: p = 0.019; ^g^ G C del/ G C del before vs G C ins/ G C ins: p = 0.012; ^h^ G C del/ G C ins before vs G C del/ G C ins after: p = 0.044; ^i^ G C del/ G C ins before vs G G del/ G C del before: p = 0.048; ^j^ G C ins/ G C ins before vs G G del/ G C del before: p = 0.036

| **Diplotype** | **A C del/**  **A C del** | | **A C del/**  **A G del** | | **A C del/**  **G C del** | | **A C del/**  **G G del** | | **A C ins/**  **A C del** | | **A C ins/**  **A C ins** | | **A C ins/**  **A T del** | | **A C ins/**  **G C del** | | **A G del/**  **A G del** | | |
| --- | --- | --- | --- | --- | --- | --- | --- | --- | --- | --- | --- | --- | --- | --- | --- | --- | --- | --- | --- |
| **Before or after IVF-ET** | **before** | **after** | **before** | **after** | **before** | **after** | **before** | **after** | **before** | **after** | **before** | **after** | **before** | **after** | **before** | **after** | **before** | **after** |  |
| Number of patients | 27 | 20 | 15 | 10 | 14 | 12 | 5 | 4 | 7 | 6 | 11 | 11 | 4 | 4 | 62 | 47 | 3 | 1 |  |
| Minimum | 1.3 | 0.0 | 0.0 | 0.0 | 0.0 | 0.0 | 17.4 | 33.8 | 42.5 | 40.76 | 35.7 | 9.6 | 2.3 | 2.1 | 1.8 | 1.3 | 70.7 | 50.8 |  |
| 25% Percentile | 30.0 | 22.4 | 8.6 | 23.9 | 39.3 | 22.3 | 18.7 | 45.2 | 47.7 | 75.50 | 42.4 | 43.7 | 2.4 | 2.1 | 46.4 | 36.1 | 70.7 | 50.8 |  |
| Median | **112.1^a, b^** | 40.3 | 52.8 | 63.1 | 70.2 | 63.8 | 57.5 | 171.5 | 61.2 | **100.2^c^** | 53.5 | 67.5 | 28.4 | 2.4 | **81.2^d, e^** | 62.2 | 74.5 | 50.8 |  |
| 75% Percentile | 220.6 | 108.6 | 127.2 | 133.4 | 121.9 | 124.0 | 77.8 | 286.0 | 83.1 | 375.9 | 105.7 | 140.3 | 218.0 | 103.6 | 271.5 | 176.1 | 142.9 | 50.8 |  |
| Maximum | 1163^i.^ | 849.5 | 258.5 | 174.8 | 436.8 | 405.3 | 92.2 | 293.5 | 113.9 | 876.9 | 565.4 | 1115 | 272.7 | 137.3 | 1492 | 1206 | 142.9 | 50. 8 |  |
| Mean | 169.7 | 134.0 | 73.7 | 76.5 | 111.8 | 95.16 | 50.1 | 167.6 | 68.4 | 235.7 | 108.7 | 172.5 | 82.9 | 36.0 | 206.1 | 169.7 | 96.0 | 50.8 |  |
| Std. Deviation | 231.3 | 218.2 | 74.4 | 62.7 | 124.6 | 107.9 | 31.5 | 130.0 | 25.0 | 319.0 | 153.7 | 317.0 | 128.8 | 67.5 | 299.9 | 254.6 | 40.7 | 0.0 |  |
| Std. Error | 44.5 | 48.8 | 19.2 | 19.8 | 33.31 | 31.2 | 14.1 | 65.0 | 9.5 | 130.2 | 46.3 | 95.6 | 64.4 | 33.8 | 38.09 | 37.1 | 23.5 | 0.0 |  |
| Lower 95% CI of mean | 78.2 | 31.9 | 32.4 | 31.7 | 39.8 | 26.61 | 10.9 | -39.3 | 45.3 | -99.13 | 5.4 | -40.5 | -122.0 | -71.4 | 129.9 | 94.9 | -4.9 | 0.0 |  |
| Upper 95% CI of mean | 261.2 | 236.2 | 114.9 | 121.4 | 183.7 | 163.7 | 89.2 | 374.5 | 91.6 | 570.5 | 211.9 | 385.4 | 287.9 | 143.4 | 282.2 | 244.4 | 197.0 | 0.0 |  |
| D'Agostino & Pearson omnibus normality test K^2^ | 43.4 | 24.1 | 5.5 | 1.7 | 13.32 | 20.6 | N too small | N too small | N too small | N too small | 29.1 | 29.2 | N too small | N too small | 53.9 | 40.2 | N too small | N too small |  |

**Supplementary Table 5** (Continued)

| **Diplotype** | **A T del/**  **A C del** | | **G C del/**  **G C del** | | **G C del/**  **G C ins** | | **G C ins/**  **G C ins** | | **G G del/**  **A C ins** | | **G G del/**  **G C del** | | **G T ins/**  **A C ins** | | **G T ins/**  **G C del** | | **G G ins/**  **G G del** | |
| --- | --- | --- | --- | --- | --- | --- | --- | --- | --- | --- | --- | --- | --- | --- | --- | --- | --- | --- |
| **Before or after IVF-ET** | **before** | **after** | **before** | **after** | **before** | **after** | **before** | **after** | **before** | **after** | **before** | **after** | **before** | **after** | **before** | **after** | **before** | **after** |
| Number of patients | 1 | 0 | 9 | 7 | 13 | 10 | 20 | 18 | 28 | 22 | 6 | 6 | 6 | 5 | 2 | 1 | 1 | 1 |
| Minimum | 51.4 | - | 43.2 | 36.9 | 0.0 | 0.0 | 2.4 | 1.8 | 0.0 | 0.0 | 6.6 | 43.2 | 16.2 | 27.9 | 218.3 | 102.6 | 57.2 | 376.1 |
| 25% Percentile | 51.4 | - | 57.3 | 53.7 | 2.4 | 49.7 | 13.4 | 31.2 | 33.1 | 25.6 | 38.3 | 46.6 | 22.7 | 30.9 | 218.3 | 102.6 | 57.2 | 376.1 |
| Median | 51.4 | - | **112.9^f, g^** | 119.3 | **28.9^h, i^** | 113.9 | **47.5^j^** | 56.3 | 63.7 | 56.7 | 301.8 | 152.4 | 44.6 | 60.6 | 250.9 | 102.6 | 57.2 | 376.1 |
| 75% Percentile | 51.4 | - | 252.8 | 303.6 | 88.2 | 350.3 | 73.9 | 99.5 | 175.8 | 149.6 | 797.5 | 340.1 | 466.1 | 1055 | 283.4 | 102.6 | 57.2 | 376.1 |
| Maximum | 51.4 | - | 1429 | 1828 | 658.8 | 968.9 | 108.5 | 249.9 | 1315 | 2122 | 1054 | 396.1 | 758.1 | 1278 | 283.4 | 102.6 | 57.2 | 376.1 |
| Mean | 51.4 | - | 267.4 | 367.3 | 88.5 | 232.7 | 47.6 | 66.6 | 139.5 | 187.0 | 404.2 | 185.6 | 209.5 | 446.4 | 250.9 | 102.6 | 57.2 | 376.1 |
| Std. Deviation | 0.0 | - | 443.0 | 650.9 | 177.2 | 303.6 | 33.4 | 58.1 | 249.7 | 445.1 | 406.9 | 152.7 | 300.9 | 577.6 | 46.0 | 0.0 | 0.0 | 0.0 |
| Std. Error | 0.0 | - | 147.7 | 246.0 | 49.2 | 96.0 | 7.5 | 13.7 | 47.2 | 94.9 | 166.1 | 62.3 | 122.8 | 258.3 | 32.5 | 0.0 | 0.0 | 0.0 |
| Lower 95% CI of mean | 0.0 | - | -73.1 | -234.7 | -18.6 | 15.5 | 31.9 | 37.7 | 42.7 | -10.3 | -22.8 | 25.3 | -106.3 | -270.8 | -162.5 | 0.00 | 0.0 | 0.0 |
| Upper 95% CI of mean | 0.0 | - | 607.9 | 969.2 | 195.6 | 449.9 | 63.2 | 95.5 | 236.3 | 384.4 | 831.2 | 345.8 | 525.3 | 1164 | 664.2 | 0. | 0. | 0. |
| D'Agostino & Pearson omnibus normality test K^2^ | N too small | N too small | 23.4 | N too small | 31.4 | 11.7 | 2.6 | 17.1 | 55.8 | 51.44 | N too small | N too small | N too small | N too small | N too small | N too small | N too small | N too small |
