# Supplementary material for: Association of Soluble HLA-G Plasma Level and HLA-G Genetic Polymorphism With Pregnancy Outcome of Patients Undergoing in vitro Fertilization Embryo Transfer
Source: Front Immunol. 2020 Jan 14;10:2982. doi: 10.3389/fimmu.2019.02982 (PMC6971053; doi:10.3389/fimmu.2019.02982)
Supplement: Supplementary file 6 [file Table_6.DOCX]

**Supplementary Table 6** HLA-G value (IU/ml) measured before and after IVF embryo transfer in patients with clinical pregnancy, depending on *HLA-G* diplotypes

Diplotypes were determined from haplotype analysis and estimated in the following order: rs1632947:-964G>A; rs1233334:-725G>C/T; rs371194629:insATTTGTTCATGCCT/del. P values are calculated by Mann-Whitney test. ^a^ A C del/ A C del after vs A C ins/ A C del after: p = 0.06; ^b^ A C del/ A G del after vs A C del/ G C del after: p = 0.029; ^c^ A C del/ G C del after vs A C ins/ A C del after: p = 0.028; ^d^ A C del/ G C del after vs A C ins/ A C ins after: p = 0.049; ^e^ A C del/ G C del after vs G G del/ G C del after: p = 0.049; ^f^ A C ins/ A C del before vs A C ins/ A C del after: p = 0.029; ^g^ A C ins/ G C del before vs G C ins/ G C ins before: p = 0.026; ^h^ G C del/ G C ins after vs G C ins/ G C ins after: p = 0.073

| **Diplotype** | **A C del/**  **A C del** | | **A C del/**  **A G del** | | **A C del/**  **G C del** | | **A C del/**  **G G del** | | **A C ins/**  **A C del** | | **A C ins/**  **A C ins** | | **A C ins/**  **A T del** | | **A C ins/**  **G C del** | |
| --- | --- | --- | --- | --- | --- | --- | --- | --- | --- | --- | --- | --- | --- | --- | --- | --- |
| **Before or after IVF-ET** | **before** | **after** | **before** | **after** | **before** | **after** | **before** | **after** | **before** | **after** | **before** | **after** | **before** | **after** | **before** | **after** |
| Number of patients | 13 | 12 | 7 | 6 | 9 | 8 | 3 | 3 | 4 | 4 | 4 | 4 | 3 | 3 | 34 | 30 |
| Minimum | 1.312 | 21.36 | 2.204 | 40.72 | 0.0 | 0.0 | 17.38 | 33.84 | 47.70 | 87.53 | 49.75 | 52.94 | 2.925 | 2.037 | 1.815 | 1.338 |
| 25% Percentile | 26.46 | 25.57 | 65.16 | 73.45 | 23.46 | 18.76 | 17.38 | 33.84 | 49.08 | 93.88 | 50.69 | 72.61 | 2.925 | 2.037 | 43.46 | 35.71 |
| Median | 44.56 | **42.50^a^** | 88.64 | **118.3^b^** | 47.82 | **42.62^c, d, e^** | 20.03 | 79.41 | **65.30^f^** | 160.9 | 80.34 | 135.9 | 53.77 | 2.182 | **92.77^g^** | 61.64 |
| 75% Percentile | 191.5 | 104.7 | 154.3 | 159.3 | 110.6 | 96.86 | 63.38 | 293.5 | 81.68 | 709.9 | 450.8 | 871.6 | 272.7 | 137.3 | 376.2 | 254.8 |
| Maximum | 391.5 | 849.5 | 162.2 | 174.8 | 337.3 | 113.3 | 63.38 | 293.5 | 83.11 | 876.9 | 565.4 | 1115 | 272.7 | 137.3 | 1492 | 1206 |
| Mean | 110.4 | 157.1 | 98.21 | 115.1 | 86.91 | 51.43 | 33.59 | 135.6 | 65.35 | 321.6 | 194.0 | 360.0 | 109.8 | 47.16 | 231.6 | 200.8 |
| Std. Deviation | 122.8 | 263.2 | 55.67 | 48.42 | 101.7 | 42.00 | 25.83 | 138.6 | 17.51 | 373.9 | 249.0 | 505.1 | 143.3 | 78.02 | 323.5 | 300.3 |
| Std. Error | 34.06 | 75.98 | 21.04 | 19.77 | 33.91 | 14.85 | 14.91 | 80.05 | 8.755 | 186.9 | 124.5 | 252.5 | 82.76 | 45.05 | 55.48 | 54.83 |
| Lower 95% CI of mean | 36.24 | -10.17 | 46.73 | 64.28 | 8.719 | 16.31 | -30.56 | -208.8 | 37.49 | -273.4 | -202.3 | -443.6 | -246.3 | -146.7 | 118.8 | 88.67 |
| Upper 95% CI of mean | 184.6 | 324.3 | 149.7 | 165.9 | 165.1 | 86.54 | 97.75 | 480.0 | 93.21 | 916.5 | 590.2 | 1164 | 465.9 | 241.0 | 344.5 | 312.9 |
| D'Agostino & Pearson omnibus normality test K^2^ | 5.571 | 15.96 | N too small | N too small | 15.40 | 1.194 | N too small | N too small | N too small | N too small | N too small | N too small | N too small | N too small | 32.44 | 23.28 |

**Supplementary Table 6** (Continued)

| **Diplotype** | **A G del/**  **A G del** | | **G C del/**  **G C del** | | **G C del/**  **G C ins** | | **G C ins/**  **G C ins** | | **G G del/**  **A C ins** | | **G G del/**  **G C del** | | **G T ins/**  **A C ins** | | **G T ins/**  **G C del** | |
| --- | --- | --- | --- | --- | --- | --- | --- | --- | --- | --- | --- | --- | --- | --- | --- | --- |
| **Before or after IVF-ET** | **before** | **after** | **before** | **after** | **before** | **after** | **before** | **after** | **before** | **after** | **before** | **after** | **before** | **after** | **before** | **after** |
| Number of patients | 1 | 1 | 7 | 6 | 6 | 6 | 11 | 10 | 21 | 16 | 3 | 3 | 5 | 4 | 1 | 1 |
| Minimum | 70.66 | 50.78 | 43.24 | 36.90 | 0.0 | 0.0 | 2.523 | 2.425 | 0.0 | 0.0 | 6.607 | 73.97 | 24.90 | 33.92 | 218.3 | 102.6 |
| 25% Percentile | 70.66 | 50.78 | 52.20 | 49.47 | 0.0 | 81.59 | 11.18 | 31.49 | 16.80 | 21.86 | 6.607 | 73.97 | 26.28 | 40.59 | 218.3 | 102.6 |
| Median | 70.66 | 50.78 | 66.28 | 90.47 | 31.21 | **132.2^h^** | 37.21 | 61.89 | 67.67 | 56.67 | 251.4 | 230.9 | 61.49 | 445.9 | 218.3 | 102.6 |
| 75% Percentile | 70.66 | 50.78 | 252.3 | 582.5 | 125.4 | 350.3 | 82.72 | 107.6 | 144.4 | 127.6 | 712.0 | 321.5 | 563.4 | 1167 | 218.3 | 102.6 |
| Maximum | 70.66 | 50.78 | 1429 | 1828 | 145.1 | 543.0 | 108.5 | 249.9 | 1315 | 2122 | 712.0 | 321.5 | 758.1 | 1278 | 218.3 | 102.6 |
| Mean | 70.66 | 50.78 | 291.5 | 377.9 | 54.38 | 200.4 | 45.83 | 77.54 | 148.5 | 203.1 | 323.3 | 208.8 | 248.2 | 551.0 | 218.3 | 102.6 |
| Std. Deviation | 0.00 | 0.00 | 506.9 | 712.3 | 62.39 | 191.3 | 37.97 | 69.60 | 284.8 | 518.2 | 358.1 | 125.2 | 319.3 | 609.8 | 0.0 | 0.00 |
| Std. Error | 0.00 | 0.00 | 191.6 | 290.8 | 25.47 | 78.09 | 11.45 | 22.01 | 62.15 | 129.5 | 206.8 | 72.30 | 142.8 | 304.9 | 0.0 | 0.00 |
| Lower 95% CI of mean | 0.00 | 0.00 | -177.3 | -369.7 | -11.10 | -0.3494 | 20.33 | 27.75 | 18.81 | -72.99 | -566.3 | -102.3 | -148.3 | -419.3 | 0.00 | 0.00 |
| Upper 95% CI of mean | 0.00 | 0.00 | 760.3 | 1125 | 119.9 | 401.1 | 71.34 | 127.3 | 278.1 | 479.3 | 1213 | 519.9 | 644.7 | 1521 | 0.00 | 0.00 |
| D'Agostino & Pearson omnibus normality test K^2^ | N too small | N too small | N too small | N too small | N too small | N too small | 2.089 | 11.94 | 44.88 | 41.16 | N too small | N too small | N too small | N too small | N too small | N too small |
