# Supplementary material for: Association of Soluble HLA-G Plasma Level and HLA-G Genetic Polymorphism With Pregnancy Outcome of Patients Undergoing in vitro Fertilization Embryo Transfer
Source: Front Immunol. 2020 Jan 14;10:2982. doi: 10.3389/fimmu.2019.02982 (PMC6971053; doi:10.3389/fimmu.2019.02982)
Supplement: Supplementary file 7 [file Table_7.DOCX]

**Supplementary Table 7** HLA-G value (IU/ml) measured before and after IVF embryo transfer in patients with a lack of pregnancy, depending on *HLA-G* diplotypes

Diplotypes were determined from haplotype analysis and estimated in the following order: rs1632947:-964G>A; rs1233334:-725G>C/T; rs371194629:insATTTGTTCATGCCT/del.

| **Diplotype** | **A C del/**  **A C del** | | **A C del/**  **A G del** | | **A C del/**  **G C del** | | **A C del/**  **G G del** | | **A C ins/**  **A C del** | | **A C ins/**  **A C ins** | | **A C ins/**  **A T del** | | **A C ins/**  **G C del** | |
| --- | --- | --- | --- | --- | --- | --- | --- | --- | --- | --- | --- | --- | --- | --- | --- | --- |
| **Before or after IVF-ET** | **before** | **after** | **before** | **after** | **before** | **after** | **before** | **after** | **before** | **after** | **before** | **after** | **before** | **after** | **before** | **after** |
| Number of patients | 5 | 3 | 2 | 1 | 4 | 3 | 1 | 0 | 3 | 2 | 2 | 2 | 1 | 1 | 12 | 5 |
| Minimum | 103.4 | 48.78 | 2.668 | 1.529 | 57.55 | 59.31 | 92.16 | - | 42.51 | 40.76 | 42.36 | 39.02 | 2.256 | 2.544 | 2.109 | 2.715 |
| 25% Percentile | 117.7 | 48.78 | 2.668 | 1.529 | 63.48 | 59.31 | 92.16 | - | 42.51 | 40.76 | 42.36 | 39.02 | 2.256 | 2.544 | 39.61 | 18.36 |
| Median | 195.5 | 109.4 | 5.618 | 1.529 | 114.6 | 138.3 | 92.16 | - | 61.21 | 63.92 | 43.30 | 42.52 | 2.256 | 2.544 | 61.74 | 42.84 |
| 75% Percentile | 314.9 | 248.1 | 8.567 | 1.529 | 364.6 | 405.3 | 92.16 | - | 113.9 | 87.08 | 44.25 | 46.02 | 2.256 | 2.544 | 222.6 | 267.1 |
| Maximum | 409.2 | 248.1 | 8.567 | 1.529 | 436.8 | 405.3 | 92.16 | - | 113.9 | 87.08 | 44.25 | 46.02 | 2.256 | 2.544 | 1357 | 268.1 |
| Mean | 212.1 | 135.4 | 5.618 | 1.529 | 180.9 | 201.0 | 92.16 | - | 72.55 | 63.92 | 43.30 | 42.52 | 2.256 | 2.544 | 203.8 | 122.8 |
| Std. Deviation | 119.8 | 102.2 | 4.171 | 0.00 | 174.8 | 181.3 | 0.00 | - | 37.03 | 32.75 | 1.339 | 4.948 | 0.00 | 0.00 | 377.2 | 132.6 |
| Std. Error | 53.58 | 58.99 | 2.950 | 0.00 | 87.41 | 104.7 | 0.00 | - | 21.38 | 23.16 | 0.9465 | 3.499 | 0.00 | 0.00 | 108.9 | 59.32 |
| Lower 95% CI of mean | 63.38 | -118.4 | -31.86 | 0.00 | -97.29 | -249.4 | 0.00 | - | -19.44 | -230.3 | 31.28 | -1.938 | 0.00 | 0.00 | -35.85 | -41.93 |
| Upper 95% CI of mean | 360.9 | 389.2 | 43.09 | 0.00 | 459.1 | 651.4 | 0.00 | - | 164.5 | 358.2 | 55.33 | 86.98 | 0.00 | 0.00 | 443.5 | 287.5 |
| D'Agostino & Pearson omnibus normality test K^2^ | N too small | N too small | N too small | N too small | N too small | N too small | N too small | N too small | N too small | N too small | N too small | N too small | N too small | N too small | 28.47 | N too small |

**Supplementary Table 7** (Continued)

| **Diplotype** | **A G del/**  **A G del** | | **A T del/**  **A C del** | | **G C del/**  **G C ins** | | **G C ins/**  **G C ins** | | **G G del/**  **A C ins** | | **G G del/**  **G C del** | | **G T ins/**  **G C del** | | **G T ins/**  **G G del** | |
| --- | --- | --- | --- | --- | --- | --- | --- | --- | --- | --- | --- | --- | --- | --- | --- | --- |
| **Before or after IVF-ET** | **before** | **after** | **before** | **after** | **before** | **after** | **before** | **after** | **before** | **after** | **before** | **after** | **before** | **after** | **before** | **after** |
| Number of patients | 2 | 0 | 1 | 0 | 3 | 2 | 2 | 1 | 3 | 3 | 2 | 2 | 1 | 0 | 1 | 1 |
| Minimum | 74.45 | - | 51.39 | - | 21.91 | 52.76 | 61.20 | 39.88 | 39.64 | 42.39 | 48.87 | 43.20 | 283.4 | - | 57.19 | 376.1 |
| 25% Percentile | 74.45 | - | 51.39 | - | 21.91 | 52.76 | 61.20 | 39.88 | 39.64 | 42.39 | 48.87 | 43.20 | 283.4 | - | 57.19 | 376.1 |
| Median | 108.7 | - | 51.39 | - | 52.29 | 510.8 | 69.90 | 39.88 | 190.9 | 69.29 | 551.6 | 45.43 | 283.4 | - | 57.19 | 376.1 |
| 75% Percentile | 142.9 | - | 51.39 | - | 658.8 | 968.9 | 78.60 | 39.88 | 211.0 | 259.1 | 1054 | 47.67 | 283.4 | - | 57.19 | 376.1 |
| Maximum | 142.9 | - | 51.39 | - | 658.8 | 968.9 | 78.60 | 39.88 | 211.0 | 259.1 | 1054 | 47.67 | 283.4 | - | 57.19 | 376.1 |
| Mean | 108.7 | - | 51.39 | - | 244.3 | 510.8 | 69.90 | 39.88 | 147.2 | 123.6 | 551.6 | 45.43 | 283.4 | - | 57.19 | 376.1 |
| Std. Deviation | 48.40 | - | 0.00 | - | 359.3 | 647.8 | 12.30 | 0.0 | 93.67 | 118.1 | 710.9 | 3.159 | 0.00 | - | 0.00 | 0.00 |
| Std. Error | 34.22 | - | 0.00 | - | 207.4 | 458.1 | 8.700 | 0.0 | 54.08 | 68.20 | 502.7 | 2.234 | 0.00 | - | 0.00 | 0.00 |
| Lower 95% CI of mean | -326.2 | - | 0.00 | - | -648.1 | -5310 | -40.65 | 0.00 | -85.51 | -169.8 | -5836 | 17.05 | 0.00 | - | 0.00 | 0.00 |
| Upper 95% CI of mean | 543.5 | - | 0.00 | - | 1137 | 6331 | 180.5 | 0.00 | 379.9 | 417.0 | 6939 | 73.81 | 0.00 | - | 0.00 | 0.00 |
| D'Agostino & Pearson omnibus normality test K^2^ | N too small | N too small | N too small | N too small | N too small | N too small | N too small | N too small | N too small | N too small | N too small | N too small | N too small | N too small | N too small | N too small |
