# Supplementary material for: Association of Soluble HLA-G Plasma Level and HLA-G Genetic Polymorphism With Pregnancy Outcome of Patients Undergoing in vitro Fertilization Embryo Transfer
Source: Front Immunol. 2020 Jan 14;10:2982. doi: 10.3389/fimmu.2019.02982 (PMC6971053; doi:10.3389/fimmu.2019.02982)
Supplement: Supplementary file 8 [file Table_8.DOCX]

**Supplementary Table 8** HLA-G value (IU/ml) measured before and after IVF embryo transfer in patients with miscarriage, depending on particular *HLA-G* diplotypes

| **Diplotype** | **A C del/**  **A C del** | | **A C del/**  **A G del** | | **A C del/**  **G C del** | | **A C del/**  **G G del** | | **A C ins/**  **A C ins** | | **A C ins/**  **G C del** | | **G C del/**  **G C del** | | **G C del/**  **G C ins** | | **G C ins/**  **G C ins** | | **G G del/**  **A C ins** | | **G G del/**  **G C del** | | **G T ins/**  **A C ins** | |
| --- | --- | --- | --- | --- | --- | --- | --- | --- | --- | --- | --- | --- | --- | --- | --- | --- | --- | --- | --- | --- | --- | --- | --- | --- |
| **Before or after IVF-ET** | **before** | **after** | **before** | **after** | **before** | **after** | **before** | **after** | **before** | **after** | **before** | **after** | **before** | **after** | **before** | **after** | **before** | **after** | **before** | **after** | **before** | **after** | **before** | **after** |
| Number of patients | 9 | 5 | 6 | 3 | 1 | 1 | 1 | 1 | 5 | 5 | 16 | 12 | 2 | 1 | 4 | 2 | 7 | 7 | 4 | 3 | 1 | 1 | 1 | 1 |
| Minimum | 2.6 | 0.0 | 0.0 | 0.0 | 59.18 | 127.6 | 57.45 | 263.6 | 35.70 | 9.637 | 2.109 | 2.710 | 112.9 | 303.6 | 2.111 | 40.58 | 2.375 | 1.776 | 31.85 | 40.22 | 352.2 | 396.1 | 16.23 | 27.88 |
| 25% Percentile | 18.7 | 1.081 | 17.40 | 0.0 | 59.18 | 127.6 | 57.45 | 263.6 | 36.36 | 26.65 | 49.23 | 38.08 | 112.9 | 303.6 | 2.240 | 40.58 | 2.461 | 3.938 | 33.05 | 40.22 | 352.2 | 396.1 | 16.23 | 27.88 |
| Median | 121.0 | 2.292 | **35.87^a^** | **31.36^b^** | 59.18 | 127.6 | 57.45 | 263.6 | 71.83 | 67.46 | **80.77^c^** | 77.72 | 183.1 | 303.6 | 15.74 | 51.48 | 47.58 | 56.50 | 39.31 | 48.27 | 352.2 | 396.1 | 16.23 | 27.88 |
| 75% Percentile | 276.3 | 192.4 | 104.2 | 41.80 | 59.18 | 127.6 | 57.45 | 263.6 | 94.11 | 125.6 | 212.2 | 116.6 | 253.3 | 303.6 | 50.39 | 62.38 | 70.02 | 98.36 | 187.4 | 405.7 | 352.2 | 396.1 | 16.23 | 27.88 |
| Maximum | 1163 | 359.8 | 258.5 | 41.80 | 59.18 | 127.6 | 57.45 | 263.6 | 105.7 | 182.7 | 503.4 | 531.1 | 253.3 | 303.6 | 57.57 | 62.38 | 74.30 | 102.8 | 235.9 | 405.7 | 352.2 | 396.1 | 16.23 | 27.88 |
| Mean | 231.7 | 77.85 | 67.71 | 24.39 | 59.18 | 127.6 | 57.45 | 263.6 | 66.55 | 74.40 | 153.4 | 111.5 | 183.1 | 303.6 | 22.79 | 51.48 | 43.93 | 54.87 | 86.60 | 164.7 | 352.2 | 396.1 | 16.23 | 27.88 |
| Std. Deviation | 364.7 | 157.9 | 95.14 | 21.76 | 0.0 | 0.0 | 0.0 | 0.0 | 30.16 | 65.08 | 163.6 | 139.8 | 99.28 | 0.0 | 26.33 | 15.41 | 30.28 | 42.80 | 99.65 | 208.7 | 0.0 | 0.0 | 0.0 | 0.0 |
| Std. Error | 121.6 | 70.63 | 38.84 | 12.56 | 0.0 | 0.0 | 0.0 | 0.0 | 13.49 | 29.11 | 40.91 | 40.36 | 70.20 | 0.0 | 13.17 | 10.90 | 11.45 | 16.18 | 49.82 | 120.5 | 0.0 | 0.0 | 0.0 | 0.0 |
| Lower 95% CI of mean | -48.6 | -118.3 | -32.14 | -29.66 | 0.0 | 0.0 | 0.0 | 0.0 | 29.11 | -6.411 | 66.22 | 22.64 | -709.0 | 0.0 | -19.11 | -87.01 | 15.92 | 15.28 | -71.96 | -353.8 | 0.0 | 0.0 | 0.0 | 0.0 |
| Upper 95% CI of mean | 512.0 | 274.0 | 167.6 | 78.43 | 0.0 | 0.0 | 0.0 | 0.0 | 104.0 | 155.2 | 240.6 | 200.3 | 1075 | 0.0 | 64.69 | 190.0 | 71.93 | 94.45 | 245.2 | 683.2 | 0.0 | 0.00 | 0.0 | 0.0 |
| D'Agostino & Pearson omnibus normality test K^2^ | 19.9 | N too small | N too small | N too small | N too small | N too small | N too small | N too small | N too small | N too small | 6.555 | 25.99 | N too small | N too small | N too small | N too small | N too small | N too small | N too small | N too small | N too small | N too small | N too small | N too small |

Diplotypes were determined from haplotype analysis and estimated in the following order: rs1632947:-964G>A; rs1233334:-725G>C/T; rs371194629:insATTTGTTCATGCCT/del. P values are calculated by Mann-Whitney test. ^a^ A C del/ A G del before vs A C ins/ G C del before: p = 0.043; ^b^ A C del/ A G del after vs A C ins/ G C del after: p = 0.05; ^c^ A C ins/ G C del before vs G C del/ G C ins before: p = 0.026
