# Supplementary material for: Association of Soluble HLA-G Plasma Level and HLA-G Genetic Polymorphism With Pregnancy Outcome of Patients Undergoing in vitro Fertilization Embryo Transfer
Source: Front Immunol. 2020 Jan 14;10:2982. doi: 10.3389/fimmu.2019.02982 (PMC6971053; doi:10.3389/fimmu.2019.02982)
Supplement: Supplementary file 9 [file Table_9.DOCX]

**Supplementary Table 9** HLA-G value (IU/ml) measured before and after IVF embryo transfer in all patients according to *HLA-G* haplotypes and depending on fresh or frozen/thawed cycle

*Haplotypes were estimated in the following order: rs1632947:-964G>A; rs1233334:-725G>C/T; rs371194629:insATTTGTTCATGCCT/del. P values are calculated by Mann-Whitney test.

Comparison of haplotypes in fresh cycle: ^a^A C del after vs A C ins after: p = 0.048; ^b^ A C ins before vs G C ins before: p = 0.025; ^c^ G C del before vs G C ins before: p = 0.014;

Comparison of haplotypes in frozen cycle: ^d^ G C ins before vs G C ins after: p = 0.02; ^e^ A C del before vs G C ins before; ^f^ A C ins before vs G C ins before: p = 0.004; ^g^ G C del before vs G C ins before: p = 0.0008; ^h^ G C ins before vs GG del before: p = 0.027; ^i^ G C ins before vs G T ins before: p = 0.052

Comparison of haplotypes G C del and G C ins (fresh vs frozen cycle) by Kruskal - Wallis test, p = 0.0001.

| **FRESH CYCLE** | | | | | | | | | | | | | | | | |
| --- | --- | --- | --- | --- | --- | --- | --- | --- | --- | --- | --- | --- | --- | --- | --- | --- |
| **Haplotype*** | **A C del** | | **A C ins** | | **A G del** | | **A T del** | | **G C del** | | **G C ins** | | **G G del** | | **G T ins** | |
| **Before or after IVF-ET** | **before** | **after** | **before** | **after** | **before** | **after** | **before** | **after** | **before** | **after** | **before** | **after** | **before** | **after** | **before** | **after** |
| Number of patients | 35 | 30 | 53 | 46 | 10 | 8 | 1 | 1 | 43 | 38 | 22 | 19 | 15 | 14 | 5 | 4 |
| Minimum | 0.0 | 0.0 | 0.0 | 0.0 | 0.0 | 0.0 | 2.256 | 2.544 | 0.0 | 0.0 | 0.0 | 0.0 | 0.0 | 0.0 | 24.90 | 33.92 |
| 25% Percentile | 23.73 | 28.62 | 39.29 | 39.60 | 6.976 | 40.99 | 2.256 | 2.544 | 38.93 | 36.90 | 15.94 | 3.938 | 27.62 | 31.61 | 26.28 | 51.09 |
| Median | 61.21 | **41.28^a^** | **53.59^b^** | 67.46 | 67.91 | 50.78 | 2.256 | 2.544 | **61.65^c^** | 61.61 | 45.94 | 40.58 | 49.98 | 51.16 | 57.19 | 239.4 |
| 75% Percentile | 154.3 | 92.24 | 94.38 | 177.8 | 104.4 | 147.2 | 2.256 | 2.544 | 137.1 | 164.1 | 72.88 | 107.5 | 110.4 | 314.7 | 139.9 | 1053 |
| Maximum | 409.2 | 876.9 | 1492 | 1278 | 162.2 | 174.8 | 2.256 | 2.544 | 1492 | 1206 | 108.5 | 543.0 | 1054 | 405.7 | 218.3 | 1278 |
| Mean | 110.0 | 84.72 | 132.4 | 214.6 | 66.10 | 79.93 | 2.256 | 2.544 | 171.8 | 150.4 | 42.85 | 87.50 | 164.9 | 143.4 | 77.91 | 447.8 |
| Std. Deviation | 124.1 | 156.7 | 237.5 | 352.1 | 57.72 | 62.96 | 0.0 | 0.0 | 295.0 | 242.1 | 33.53 | 132.4 | 302.2 | 152.8 | 80.24 | 573.2 |
| Std. Error | 20.98 | 28.61 | 32.62 | 51.92 | 18.25 | 22.26 | 0.0 | 0.0 | 44.99 | 39.27 | 7.148 | 30.36 | 78.04 | 40.84 | 35.88 | 286.6 |
| Lower 95% CI of mean | 67.35 | 26.20 | 66.96 | 110.0 | 24.81 | 27.29 | 0.0 | 0.0 | 81.00 | 70.86 | 27.99 | 23.71 | -2.499 | 55.17 | -21.72 | -464.3 |
| Upper 95% CI of mean | 152.6 | 143.2 | 197.9 | 319.1 | 107.4 | 132.6 | 0.0 | 0.0 | 262.6 | 230.0 | 57.72 | 151.3 | 332.2 | 231.6 | 177.5 | 1360 |
| D'Agostino & Pearson omnibus normality test K^2^ | 9.413 | 64.51 | 77.83 | 29.48 | 0.8668 | 1.447 | N too small | N too small | 49.99 | 48.45 | 1.419 | 27.54 | 21.43 | 4.049 | N too small | N too small |

**Supplementary Table 9** (Continued)

| **FROZEN CYCLE** | | | | | | | | | | | | | | | | |
| --- | --- | --- | --- | --- | --- | --- | --- | --- | --- | --- | --- | --- | --- | --- | --- | --- |
| **Haplotype*** | **A C del** | | **A C ins** | | **A G del** | | **A T del** | | **G C del** | | **G C ins** | | **G G del** | | **G T ins** | |
| **Before or after IVF-ET** | **before** | **after** | **before** | **after** | **before** | **after** | **before** | **after** | **before** | **after** | **before** | **after** | **before** | **after** | **before** | **after** |
| Number of patients | 61 | 42 | 76 | 60 | 11 | 4 | 4 | 3 | 72 | 52 | 31 | 27 | 25 | 19 | 4 | 3 |
| Minimum | 1.312 | 0.0 | 0.0 | 1.338 | 2.668 | 1.529 | 2.925 | 2.037 | 0.0 | 1.338 | 0.0 | 1.776 | 0.0 | 3.551 | 16.23 | 27.88 |
| 25% Percentile | 34.66 | 24.24 | 44.81 | 37.61 | 32.18 | 8.986 | 15.04 | 2.037 | 46.00 | 43.41 | 11.18 | 56.14 | 36.90 | 43.20 | 83.02 | 27.88 |
| Median | 88.64 | 93.94 | 84.61 | 61.39 | 74.45 | 57.86 | 52.58 | 2.182 | 100.9 | 84.41 | **47.58^d, e, f, g, h, i^** | 79.30 | 84.02 | 73.97 | 326.0 | 60.61 |
| 75% Percentile | 191.0 | 248.1 | 209.9 | 139.5 | 142.9 | 103.7 | 218.0 | 137.3 | 257.8 | 267.6 | 74.30 | 108.1 | 208.8 | 230.9 | 660.8 | 831.1 |
| Maximum | 1163 | 849.5 | 1357 | 2122 | 258.5 | 110.1 | 272.7 | 137.3 | 1429 | 1828 | 658.8 | 968.9 | 1315 | 2122 | 758.1 | 831.1 |
| Mean | 143.7 | 162.2 | 185.8 | 163.4 | 92.72 | 56.83 | 95.20 | 47.16 | 220.1 | 232.4 | 68.08 | 113.5 | 166.6 | 224.6 | 356.6 | 306.5 |
| Std. Deviation | 213.0 | 211.4 | 268.9 | 314.1 | 72.53 | 49.33 | 120.6 | 78.02 | 312.3 | 381.2 | 116.0 | 179.5 | 264.2 | 471.9 | 306.9 | 454.6 |
| Std. Error | 27.27 | 32.62 | 30.84 | 40.55 | 21.87 | 24.67 | 60.32 | 45.05 | 36.81 | 52.86 | 20.84 | 34.54 | 52.83 | 108.3 | 153.4 | 262.5 |
| Lower 95% CI of mean | 89.11 | 96.28 | 124.4 | 82.26 | 43.99 | -21.67 | -96.75 | -146.7 | 146.7 | 126.3 | 25.53 | 42.46 | 57.58 | -2.853 | -131.7 | -822.8 |
| Upper 95% CI of mean | 198.2 | 228.0 | 247.3 | 244.5 | 141.4 | 135.3 | 287.1 | 241.0 | 293.5 | 338.5 | 110.6 | 184.4 | 275.7 | 452.0 | 845.0 | 1436 |
| D'Agostino & Pearson omnibus normality test K^2^ | 77.23 | 27.42 | 63.80 | 92.26 | 4.523 | N too small | N too small | N too small | 57.35 | 57.44 | 64.61 | 58.58 | 47.38 | 45.61 | N too small | N too small |
