# Supplementary material for: Association of Soluble HLA-G Plasma Level and HLA-G Genetic Polymorphism With Pregnancy Outcome of Patients Undergoing in vitro Fertilization Embryo Transfer
Source: Front Immunol. 2020 Jan 14;10:2982. doi: 10.3389/fimmu.2019.02982 (PMC6971053; doi:10.3389/fimmu.2019.02982)
Supplement: Supplementary file 10 [file Table_10.DOCX]

**Supplementary Table 10** HLA-G value (IU/ml) measured before and after IVF embryo transfer in patients according to *HLA-G* diplotypes and depending on fresh or frozen/thawed cycle

Diplotypes were determined from haplotype analysis and estimated in the following order: rs1632947:-964G>A; rs1233334:-725G>C/T; rs371194629:insATTTGTTCATGCCT/del. P values are calculated by Mann-Whitney test.

Comparison of diplotypes in fresh cycle: ^a^ A C del/ A C del after vs A C ins/ A C del after: p = 0.038; ^b^ A C ins/ A C del before vs G C del/ G C ins before: p = 0.05; ^c^ A C ins / A C ins before vs G C del/ G C ins before: p = 0.035; ^d^ A C ins/ G C del before vs G C del/ G C ins before: p = 0.019; ^e^ G C del/ G C del before vs G C del/ G C ins before: p = 0.038

Comparison of diplotypes in frozen cycle: ^f^ A C del/ A G del after vs G C del/ G C ins after: p = 0.032; ^g^ A C del/ G C del before vs G C ins/ G C ins before: p = 0.034; ^h^ A C del/ G G del after vs G C ins/ G C ins after: p = 0.038; ^i^ A C ins/ G C del before vs G C ins/ G C ins before: p = 0.027; ^j^ G C del/ G C del before vs G C ins/ G C ins before: p = 0.018; ^k^ G C del/ G C ins after vs G C ins/ G C ins after: p = 0.0022; ^l^ G C del/ G C ins after vs G G del/ A C ins after: p = 0.03

Comparison of diplotypes G C del/ G C del, G C del/ G C ins and G C ins/ G C ins (fresh vs frozen cycle) by Kruskal - Wallis test, p = 0.014

| **FRESH CYCLE** | | | | | | | | | | | | | | | | |
| --- | --- | --- | --- | --- | --- | --- | --- | --- | --- | --- | --- | --- | --- | --- | --- | --- |
| **Diplotype** | **A C del/**  **A C del** | | **A C del/**  **A G del** | | **A C del/**  **G C del** | | **A C del/**  **G G del** | | **A C ins/**  **A C del** | | **A C ins/**  **A C ins** | | **A C ins/**  **A T del** | | **A C ins/**  **G C del** | |
| **Before or after IVF-ET** | **before** | **after** | **before** | **after** | **before** | **after** | **before** | **after** | **before** | **after** | **before** | **after** | **before** | **after** | **before** | **after** |
| Number of patients | 7 | 6 | 8 | 6 | 6 | 6 | 2 | 2 | 5 | 4 | 7 | 7 | 1 | 1 | 20 | 16 |
| Minimum | 2.676 | 2.162 | 0.0 | 0.0 | 0.0 | 0.0 | 20.03 | 33.84 | 42.51 | 40.76 | 35.70 | 9.637 | 2.256 | 2.544 | 2.109 | 4.795 |
| 25% Percentile | 30.04 | 19.30 | 3.795 | 30.54 | 17.39 | 13.82 | 20.03 | 33.84 | 45.11 | 52.46 | 42.36 | 46.02 | 2.256 | 2.544 | 42.07 | 37.78 |
| Median | **132.0^a^** | 31.37 | 52.36 | 84.11 | 35.77 | 49.09 | 41.70 | 56.63 | **61.21^b^** | 100.2 | **71.83^c^** | 68.59 | 2.256 | 2.544 | **64.66^d^** | 57.20 |
| 75% Percentile | 301.2 | 53.28 | 137.7 | 159.3 | 145.3 | 114.3 | 63.38 | 79.41 | 80.25 | 685.9 | 105.7 | 182.7 | 2.256 | 2.544 | 210.5 | 151.1 |
| Maximum | 409.2 | 66.78 | 162.2 | 174.8 | 337.3 | 138.3 | 63.38 | 79.41 | 83.11 | 876.9 | 565.4 | 1115 | 2.256 | 2.544 | 1492 | 1206 |
| Mean | 173.0 | 34.25 | 64.96 | 89.65 | 85.55 | 60.21 | 41.70 | 56.63 | 62.39 | 279.5 | 136.2 | 231.6 | 2.256 | 2.544 | 205.9 | 190.4 |
| Std. Deviation | 162.3 | 21.91 | 65.40 | 71.39 | 126.4 | 54.01 | 30.65 | 32.23 | 17.80 | 399.4 | 190.8 | 393.8 | 0.0 | 0.0 | 342.7 | 340.8 |
| Std. Error | 61.33 | 8.947 | 23.12 | 29.15 | 51.59 | 22.05 | 21.67 | 22.79 | 7.959 | 199.7 | 72.13 | 148.9 | 0.0 | 0.0 | 76.63 | 85.20 |
| Lower 95% CI of mean | 22.90 | 11.25 | 10.29 | 14.73 | -47.07 | 3.527 | -233.7 | -232.9 | 40.29 | -355.9 | -40.33 | -132.6 | 0.0 | 0.0 | 45.47 | 8.773 |
| Upper 95% CI of mean | 323.0 | 57.24 | 119.6 | 164.6 | 218.2 | 116.9 | 317.1 | 346.2 | 84.48 | 915.0 | 312.7 | 595.8 | 0.00 | 0.00 | 366.3 | 372.0 |
| D'Agostino & Pearson omnibus normality test K^2^ | N too small | N too small | 1.407 | N too small | N too small | N too small | N too small | N too small | N too small | N too small | N too small | N too small | N too small | N too small | 35.15 | 22.57 |

| **FRESH CYCLE** | | | | | | | | | | | | | | | | | | |
| --- | --- | --- | --- | --- | --- | --- | --- | --- | --- | --- | --- | --- | --- | --- | --- | --- | --- | --- |
| **Diplotype** | **A G del/**  **A G del** | | **G C del/**  **G C del** | | **G C del/**  **G C ins** | | **G C ins/**  **G C ins** | | **G G del/**  **A C ins** | | **G G del/**  **G C del** | | **G T ins/**  **A C ins** | | **G T ins/**  **G C del** | | **G T ins/**  **G G del** | |
| **Before or after IVF-ET** | **before** | **after** | **before** | **after** | **before** | **after** | **before** | **after** | **before** | **after** | **before** | **after** | **before** | **after** | **before** | **after** | **before** | **after** |
| Number of patients | 1 | 1 | 4 | 4 | 6 | 5 | 8 | 7 | 10 | 9 | 2 | 2 | 3 | 2 | 1 | 1 | 1 | 1 |
| Minimum | 70.66 | 50.78 | 52.20 | 36.90 | 0.0 | 0.0 | 2.375 | 2.425 | 0.0 | 0.0 | 712.0 | 47.67 | 24.90 | 33.92 | 218.3 | 102.6 | 57.19 | 376.1 |
| 25% Percentile | 70.66 | 50.78 | 54.73 | 43.08 | 16.43 | 20.29 | 7.472 | 3.938 | 22.21 | 13.63 | 712.0 | 47.67 | 24.90 | 33.92 | 218.3 | 102.6 | 57.19 | 376.1 |
| Median | 70.66 | 50.78 | **64.29^e^** | 114.4 | 26.45 | 52.76 | 46.64 | 35.01 | 42.54 | 49.13 | 883.1 | 184.6 | 27.65 | 656.2 | 218.3 | 102.6 | 57.19 | 376.1 |
| 75% Percentile | 70.66 | 50.78 | 101.2 | 269.5 | 53.61 | 302.7 | 77.17 | 107.5 | 72.32 | 285.7 | 1054 | 321.5 | 61.49 | 1278 | 218.3 | 102.6 | 57.19 | 376.1 |
| Maximum | 70.66 | 50.78 | 112.9 | 303.6 | 57.57 | 543.0 | 108.5 | 249.9 | 190.9 | 405.7 | 1054 | 321.5 | 61.49 | 1278 | 218.3 | 102.6 | 57.19 | 376.1 |
| Mean | 70.66 | 50.78 | 73.41 | 142.3 | 30.78 | 139.8 | 47.38 | 68.84 | 56.63 | 127.7 | 883.1 | 184.6 | 38.01 | 656.2 | 218.3 | 102.6 | 57.19 | 376.1 |
| Std. Deviation | 0.0 | 0.0 | 26.96 | 121.5 | 21.24 | 226.7 | 37.94 | 87.20 | 56.18 | 154.2 | 242.1 | 193.6 | 20.38 | 880.0 | 0.0 | 0.0 | 0.0 | 0.0 |
| Std. Error | 0.0 | 0.0 | 13.48 | 60.73 | 8.671 | 101.4 | 13.41 | 32.96 | 17.77 | 51.40 | 171.2 | 136.9 | 11.77 | 622.3 | 0.0 | 0.0 | 0.0 | 0.0 |
| Lower 95% CI of mean | 0.0 | 0.0 | 30.51 | -50.95 | 8.491 | -141.7 | 15.66 | -11.81 | 16.44 | 9.147 | -1292 | -1555 | -12.61 | -7251 | 0.0 | 0.0 | 0.0 | 0.0 |
| Upper 95% CI of mean | 0.0 | 0.0 | 116.3 | 335.6 | 53.07 | 421.2 | 79.10 | 149.5 | 96.81 | 246.2 | 3058 | 1924 | 88.64 | 8563 | 0.0 | 0.0 | 0.0 | 0.0 |
| D'Agostino & Pearson omnibus normality test K^2^ | N too small | N too small | N too small | N too small | N too small | N too small | 0.4989 | N too small | 9.988 | 2.237 | N too small | N too small | N too small | N too small | N too small | N too small | N too small | N too small |

**Supplementary Table 10** (Continued)

**Supplementary Table 10** (Continued)

| **FROZEN CYCLE** | | | | | | | | | | | | | | | | |
| --- | --- | --- | --- | --- | --- | --- | --- | --- | --- | --- | --- | --- | --- | --- | --- | --- |
| **Diplotype** | **A C del/**  **A C del** | | **A C del/**  **A G del** | | **A C del/**  **G C del** | | **A C del/**  **G G del** | | **A C ins/**  **A C del** | | **A C ins/**  **A C ins** | | **A C ins/**  **A T del** | | **A C ins/**  **G C del** | |
| **Before or after IVF-ET** | **before** | **after** | **before** | **after** | **before** | **after** | **before** | **after** | **before** | **after** | **before** | **after** | **before** | **after** | **before** | **after** |
| Number of patients | 20 | 14 | 7 | 4 | 8 | 6 | 3 | 2 | 2 | 2 | 4 | 4 | 3 | 3 | 42 | 31 |
| Minimum | 1.312 | 0.0 | 2.668 | 1.529 | 44.42 | 19.76 | 17.38 | 263.6 | 53.19 | 87.08 | 37.01 | 39.02 | 2.925 | 2.037 | 1.815 | 1.338 |
| 25% Percentile | 30.34 | 21.49 | 23.20 | 8.986 | 57.96 | 46.50 | 17.38 | 263.6 | 53.19 | 87.08 | 38.82 | 40.18 | 2.925 | 2.037 | 46.36 | 34.00 |
| Median | 107.8 | 76.97 | 52.78 | **57.86^f^** | **96.24^g^** | 86.30 | 57.45 | **278.5^h^** | 83.56 | 148.0 | 48.87 | 48.30 | 53.77 | 2.182 | **92.77^i^** | 66.21 |
| 75% Percentile | 194.6 | 276.0 | 127.2 | 103.7 | 139.3 | 197.0 | 92.16 | 293.5 | 113.9 | 208.9 | 93.77 | 118.4 | 272.7 | 137.3 | 315.8 | 266.2 |
| Maximum | 1163 | 849.5 | 258.5 | 110.1 | 436.8 | 405.3 | 92.16 | 293.5 | 113.9 | 208.9 | 107.2 | 140.3 | 272.7 | 137.3 | 1357 | 770.4 |
| Mean | 168.5 | 176.8 | 83.61 | 56.83 | 131.5 | 130.1 | 55.66 | 278.5 | 83.56 | 148.0 | 60.49 | 68.97 | 109.8 | 47.16 | 206.2 | 159.0 |
| Std. Deviation | 254.8 | 250.7 | 87.87 | 49.33 | 128.1 | 140.6 | 37.42 | 21.17 | 42.94 | 86.17 | 31.86 | 47.88 | 143.3 | 78.02 | 281.8 | 202.4 |
| Std. Error | 56.97 | 67.01 | 33.21 | 24.67 | 45.28 | 57.38 | 21.61 | 14.97 | 30.36 | 60.93 | 15.93 | 23.94 | 82.76 | 45.05 | 43.48 | 36.35 |
| Lower 95% CI of mean | 49.30 | 32.02 | 2.339 | -21.67 | 24.37 | -17.41 | -37.31 | 88.36 | -302.2 | -626.2 | 9.789 | -7.216 | -246.3 | -146.7 | 118.4 | 84.78 |
| Upper 95% CI of mean | 287.8 | 321.5 | 164.9 | 135.3 | 238.5 | 277.6 | 148.6 | 468.7 | 469.3 | 922.2 | 111.2 | 145.2 | 465.9 | 241.0 | 294.0 | 233.3 |
| D'Agostino & Pearson omnibus normality test K^2^ | 39.69 | 12.97 | N too small | N too small | 17.63 | N too small | N too small | N too small | N too small | N too small | N too small | N too small | N too small | N too small | 37.99 | 18.03 |

| **FROZEN CYCLE** | | | | | | | | | | | | | | | | | | |
| --- | --- | --- | --- | --- | --- | --- | --- | --- | --- | --- | --- | --- | --- | --- | --- | --- | --- | --- |
| **Diplotype** | **A G del/**  **A G del** | | **A T del/**  **A C del** | | **G C del/**  **G C del** | | **G C del/**  **G C ins** | | **G C ins/**  **G C ins** | | **G G del/**  **A C ins** | | **G G del/**  **G C del** | | **G T ins/**  **A C ins** | | **G T ins/**  **G C del** | |
| **Before or after IVF-ET** | **before** | **after** | **before** | **after** | **before** | **after** | **before** | **after** | **before** | **after** | **before** | **after** | **before** | **after** | **before** | **after** | **before** | **after** |
| Number of patients | 2 | 0 | 1 | 0 | 5 | 3 | 7 | 5 | 12 | 11 | 18 | 13 | 4 | 4 | 3 | 3 | 1 | 0 |
| Minimum | 74.45 | - | 51.39 | - | 43.24 | 53.66 | 0.0 | 108.8 | 2.461 | 1.776 | 0.0 | 3.551 | 6.607 | 43.20 | 16.23 | 27.88 | 283.4 | - |
| 25% Percentile | 74.45 | - | 51.39 | - | 89.19 | 53.66 | 2.111 | 113.9 | 14.50 | 39.88 | 39.43 | 33.00 | 17.17 | 50.89 | 16.23 | 27.88 | 283.4 | - |
| Median | 108.7 | - | 51.39 | - | **252.3^j^** | 119.3 | 38.37 | **145.3^k. l^** | 54.39 | 67.64 | 93.52 | 62.90 | 150.2 | 152.4 | 368.7 | 60.61 | 283.4 | - |
| 75% Percentile | 142.9 | - | 51.39 | - | 841.1 | 1828 | 145.1 | 627.5 | 73.23 | 98.36 | 207.8 | 119.2 | 327.0 | 354.8 | 758.1 | 831.1 | 283.4 | - |
| Maximum | 142.9 | - | 51.39 | - | 1429 | 1828 | 658.8 | 968.9 | 93.95 | 108.1 | 1315 | 2122 | 352.2 | 396.1 | 758.1 | 831.1 | 283.4 | - |
| Mean | 108.7 | - | 51.39 | - | 422.6 | 667.2 | 138.0 | 325.6 | 47.70 | 65.23 | 185.5 | 228.1 | 164.8 | 186.0 | 381.0 | 306.5 | 283.4 | - |
| Std. Deviation | 48.40 | - | 0.0 | - | 569.4 | 1006 | 237.1 | 366.6 | 31.79 | 34.32 | 301.7 | 571.3 | 164.4 | 162.4 | 371.1 | 454.6 | 0.0 | - |
| Std. Error | 34.22 | - | 0.0 | - | 254.6 | 581.0 | 89.63 | 163.9 | 9.177 | 10.35 | 71.12 | 158.5 | 82.20 | 81.18 | 214.2 | 262.5 | 0.0 | - |
| Lower 95% CI of mean | -326.2 | - | 0.0 | - | -284.4 | -1833 | -81.35 | -129.6 | 27.50 | 42.17 | 35.48 | -117.1 | -96.83 | -72.32 | -540.8 | -822.8 | 0.0 | - |
| Upper 95% CI of mean | 543.5 | - | 0.0 | - | 1130 | 3167 | 357.3 | 780.8 | 67.90 | 88.29 | 335.6 | 573.4 | 426.4 | 444.4 | 1303 | 1436 | 0.0 | - |
| D'Agostino & Pearson omnibus normality test K^2^ | N too small | N too small | N too small | N too small | N too small | N too small | N too small | N too small | 2.049 | 0.8196 | 37.97 | 35.49 | N too small | N too small | N too small | N too small | N too small | N too small |

**Supplementary Table 10** (Continued)
