# Supplementary material for: Association of Soluble HLA-G Plasma Level and HLA-G Genetic Polymorphism With Pregnancy Outcome of Patients Undergoing in vitro Fertilization Embryo Transfer
Source: Front Immunol. 2020 Jan 14;10:2982. doi: 10.3389/fimmu.2019.02982 (PMC6971053; doi:10.3389/fimmu.2019.02982)
Supplement: Supplementary file 11 [file Table_11.DOCX]

| **SHORT PROTOCOL** | | | | | | | | | | | | | | | | |
| --- | --- | --- | --- | --- | --- | --- | --- | --- | --- | --- | --- | --- | --- | --- | --- | --- |
| **Haplotype*** | **A C del** | | **A C ins** | | **A G del** | | **A T del** | | **G C del** | | **G C ins** | | **G G del** | | **G T ins** | |
| **Before or after IVF-ET** | **before** | **after** | **before** | **after** | **before** | **after** | **before** | **after** | **before** | **after** | **before** | **after** | **before** | **after** | **before** | **after** |
| Number of patients | 70 | 53 | 91 | 75 | 15 | 10 | 5 | 4 | 91 | 74 | 43 | 36 | 27 | 23 | 6 | 5 |
| Minimum | 0.0 | 0.0 | 0.0 | 0.0 | 0.0 | 0.0 | 2.256 | 2.037 | 0.0 | 0.0 | 0.0 | 0.0 | 0.0 | 0.0 | 24.90 | 33.92 |
| 25% Percentile | 27.68 | 28.35 | 44.68 | 39.10 | 23.20 | 38.38 | 2.591 | 2.073 | 47.82 | 43.11 | 3.239 | 25.66 | 20.03 | 40.22 | 26.96 | 47.27 |
| Median | **58.25^c, e^** | 66.78 | **65.72^b^** | 62.90 | 65.16 | 50.78 | 51.39 | **2.363^d^** | **67.66^a^** | **84.41^f^** | 47.58 | 56.50 | 58.93 | 62.90 | 139.9 | 102.6 |
| 75% Percentile | 163.5 | 113.1 | 155.7 | 176.1 | 88.64 | 114.2 | 163.2 | 103.6 | 252.3 | 241.6 | 78.60 | 107.5 | 235.9 | 293.5 | 466.1 | 1055 |
| Maximum | 1163 | 876.9 | 1492 | 2122 | 258.5 | 154.2 | 272.7 | 137.3 | 1492 | 1828 | 658.8 | 968.9 | 1315 | 2122 | 758.1 | 1278 |
| Mean | 129.7 | 133.3 | 164.5 | 196.2 | 72.10 | 69.05 | 76.61 | 36.00 | 209.2 | 219.6 | 60.54 | 111.3 | 199.3 | 218.5 | 243.2 | 461.4 |
| Std. Deviation | 207.7 | 202.0 | 261.1 | 351.9 | 67.96 | 48.14 | 112.4 | 67.50 | 311.9 | 356.3 | 101.4 | 180.3 | 328.4 | 435.8 | 285.9 | 564.9 |
| Std. Error | 24.82 | 27.74 | 27.37 | 40.63 | 17.55 | 15.22 | 50.28 | 33.75 | 32.70 | 41.42 | 15.46 | 30.05 | 63.20 | 90.86 | 116.7 | 252.6 |
| Lower 95% CI of mean | 80.13 | 77.64 | 110.1 | 115.2 | 34.46 | 34.61 | -63.00 | -71.40 | 144.2 | 137.0 | 29.35 | 50.27 | 69.45 | 30.09 | -56.81 | -240.1 |
| Upper 95% CI of mean | 179.2 | 189.0 | 218.8 | 277.2 | 109.7 | 103.5 | 216.2 | 143.4 | 274.1 | 302.1 | 91.74 | 172.3 | 329.3 | 407.0 | 543.2 | 1163 |
| D'Agostino & Pearson omnibus normality test K^2^ | 83.98 | 49.64 | 83.96 | 78.12 | 10.94 | 0.7725 | N too small | N too small | 67.85 | 71.58 | 82.29 | 55.05 | 27.61 | 50.49 | N too small | N too small |

**Supplementary Table 11** HLA-G value (IU/ml) measured before and after IVF embryo transfer in all patients according to particular *HLA-G* haplotypes and depending on short antagonist or long agonist ovarian stimulation protocol

*Haplotypes were estimated in the following order: rs1632947:-964G>A; rs1233334:-725G>C/T; rs371194629:ins ATTTGTTCATGCCT/del.

For short protocol: ^a^ G C del before vs G C ins before: p = 0.0005; ^b^ A C ins before vs G C ins before: p = 0.0046; ^c^ A C del before vs G C ins before: p = 0.024; ^d^ A T del after vs G C del after: p = 0.04

For short vs long protocol: ^e^ A C del before: p = 0.078; ^f^ G C del after: p = 0.08

Comparison of haplotypes G C del and G C ins (short vs long cycle) by Kruskal - Wallis test, p = 0.001

**Supplementary Table 11** (Continued)

| **LONG PROTOCOL** | | | | | | | | | | | | | | |
| --- | --- | --- | --- | --- | --- | --- | --- | --- | --- | --- | --- | --- | --- | --- |
| **Haplotype*** | **A C del** | | **A C ins** | | **A G del** | | **G C del** | | **G C ins** | | **G G del** | | **G T ins** | |
| **Before or after IVF-ET** | **before** | **after** | **before** | **after** | **before** | **after** | **before** | **after** | **before** | **after** | **before** | **after** | **before** | **after** |
| Number of patients | 26 | 19 | 37 | 31 | 6 | 2 | 23 | 16 | 10 | 10 | 13 | 10 | 3 | 2 |
| Minimum | 0.0 | 0.0 | 0.0 | 2.334 | 2.668 | 1.529 | 0.0 | 0.0 | 24.46 | 34.60 | 0.0 | 2.334 | 16.23 | 27.88 |
| 25% Percentile | 58.64 | 18.43 | 39.73 | 36.10 | 56.50 | 1.529 | 39.81 | 33.61 | 34.03 | 34.91 | 42.54 | 38.24 | 16.23 | 27.88 |
| Median | **105.7^h^** | 31.86 | 83.11 | 61.12 | **108.7^g^** | **88.17^i^** | 85.20 | 53.66 | 45.94 | 79.30 | 63.38 | 81.53 | 57.19 | 202.0 |
| 75% Percentile | 176.8 | 174.8 | 178.0 | 140.3 | 147.7 | 174.8 | 171.3 | 107.7 | 53.01 | 104.2 | 167.0 | 207.8 | 283.4 | 376.1 |
| Maximum | 391.5 | 551.0 | 1357 | 1115 | 162.2 | 174.8 | 1357 | 531.1 | 70.02 | 108.1 | 206.7 | 376.1 | 283.4 | 376.1 |
| Mean | 136.1 | 120.4 | 165.3 | 160.0 | 99.92 | 88.17 | 180.0 | 97.02 | 45.00 | 71.97 | 96.63 | 124.9 | 118.9 | 202.0 |
| Std. Deviation | 108.6 | 170.9 | 252.6 | 275.5 | 60.54 | 122.5 | 290.0 | 132.2 | 15.74 | 33.58 | 69.15 | 118.4 | 143.9 | 246.2 |
| Std. Error | 21.30 | 39.21 | 41.53 | 49.49 | 24.71 | 86.64 | 60.46 | 33.05 | 4.977 | 10.62 | 19.18 | 37.44 | 83.07 | 174.1 |
| Lower 95% CI of mean | 92.21 | 38.00 | 81.09 | 58.91 | 36.39 | -1013 | 54.59 | 26.58 | 33.74 | 47.95 | 54.84 | 40.18 | -238.5 | -2010 |
| Upper 95% CI of mean | 179.9 | 202.7 | 249.5 | 261.0 | 163.5 | 1189 | 305.4 | 167.5 | 56.26 | 95.99 | 138.4 | 209.6 | 476.4 | 2414 |
| D'Agostino & Pearson omnibus normality test K^2^ | 6.418 | 14.38 | 51.73 | 39.01 | N too small | N too small | 41.13 | 27.10 | 0.4922 | 6.124 | 2.585 | 3.886 | N too small | N too small |

For long protocol: ^g^ A G del before vs G C ins before: p = 0.03; ^h^ A C del before vs G C ins before: p = 0.0076; ^i^ A G del after vs G T ins after: p < 0.0001
