# Supplementary material for: Association of Soluble HLA-G Plasma Level and HLA-G Genetic Polymorphism With Pregnancy Outcome of Patients Undergoing in vitro Fertilization Embryo Transfer
Source: Front Immunol. 2020 Jan 14;10:2982. doi: 10.3389/fimmu.2019.02982 (PMC6971053; doi:10.3389/fimmu.2019.02982)
Supplement: Supplementary file 12 [file Table_12.DOCX]

**Supplementary Table 12** HLA-G value (IU/ml) measured before and after IVF – embryo transfer in patients according to *HLA-G* diplotypes depending on short (with GnRH-antagonist) or long (with GnRH-agonist) ovarian stimulation protocol.

| **SHORT PROTOCOL** | | | | | | | | | | | | | | | | |
| --- | --- | --- | --- | --- | --- | --- | --- | --- | --- | --- | --- | --- | --- | --- | --- | --- |
| **Diplotype** | **A C del/**  **A C del** | | **A C del/**  **A G del** | | **A C del/**  **G C del** | | **A C del/**  **G G del** | | **A C ins/**  **A C del** | | **A C ins/**  **A C ins** | | **A C ins/**  **A T del** | | **A C ins/**  **G C del** | |
| **Before or after IVF-ET** | **before** | **after** | **before** | **after** | **before** | **after** | **before** | **after** | **before** | **after** | **before** | **after** | **before** | **after** | **before** | **after** |
| Number of patients | 20 | 15 | 13 | 8 | 7 | 7 | 3 | 3 | 6 | 5 | 5 | 5 | 4 | 4 | 48 | 38 |
| Minimum | 1.312 | 2.162 | 0.0 | 0.0 | 23.73 | 29.82 | 17.38 | 33.84 | 42.51 | 40.76 | 37.01 | 43.66 | 2.256 | 2.037 | 1.815 | 1.338 |
| 25% Percentile | 25.25 | 21.53 | 15.88 | 33.70 | 47.82 | 59.31 | 17.38 | 33.84 | 46.40 | 63.92 | 43.38 | 48.30 | 2.423 | 2.073 | 46.86 | 35.71 |
| Median | 63.98 | 48.78 | 52.78 | 63.08 | **59.18^a^** | 106.4 | 20.03 | 263.6 | 57.20 | 112.9 | 71.83 | 67.46 | 28.35 | 2.363 | **73.38^b, c^** | 64.19 |
| 75% Percentile | 194.6 | 106.0 | 107.9 | 122.3 | 337.3 | 127.6 | 57.45 | 293.5 | 86.53 | 542.9 | 94.88 | 157.1 | 218.0 | 103.6 | 286.9 | 190.5 |
| Maximum | 1163 | 849.5 | 258.5 | 154.2 | 436.8 | 405.3 | 57.45 | 293.5 | 113.9 | 876.9 | 107.2 | 182.7 | 272.7 | 137.3 | 1492 | 1206 |
| Mean | 162.9 | 121.6 | 72.32 | 73.61 | 153.7 | 130.0 | 31.62 | 197.0 | 65.99 | 265.3 | 69.67 | 95.67 | 82.91 | 36.00 | 196.6 | 178.0 |
| Std. Deviation | 261.3 | 219.7 | 73.41 | 53.48 | 164.2 | 126.1 | 22.41 | 142.1 | 26.45 | 347.3 | 27.58 | 59.56 | 128.8 | 67.50 | 284.3 | 272.5 |
| Std. Error | 58.43 | 56.71 | 20.36 | 18.91 | 62.06 | 47.68 | 12.94 | 82.02 | 10.80 | 155.3 | 12.33 | 26.63 | 64.40 | 33.75 | 41.04 | 44.20 |
| Lower 95% CI of mean | 40.65 | -0.01133 | 27.96 | 28.90 | 1.797 | 13.34 | -24.05 | -155.9 | 38.23 | -166.0 | 35.43 | 21.72 | -122.0 | -71.40 | 114.1 | 88.39 |
| Upper 95% CI of mean | 285.2 | 243.3 | 116.7 | 118.3 | 305.5 | 246.7 | 87.28 | 549.9 | 93.75 | 696.6 | 103.9 | 169.6 | 287.9 | 143.4 | 279.2 | 267.5 |
| D'Agostino & Pearson omnibus normality test K^2^ | 37.23 | 30.02 | 8.398 | 0.9030 | N too small | N too small | N too small | N too small | N too small | N too small | N too small | N too small | N too small | N too small | 48.17 | 33.30 |

Diplotypes were determined from haplotypes analysis and estimated in the following order: rs1632947:-964G>A; rs1233334:-725G>C/T; rs371194629:insATTTGTTCATGCCT/del. P values are calculated by Mann-Whitney test.

Comparison of diplotypes in short protocol: ^a^ A C del/ G C del before vs G C del/ G C del before: p = 0.02; ^b^ A C ins/ G C del before vs G C del/ G C ins before: p = 0.02; ^c^ A C ins/ G C del before vs G C ins/ G C ins before: p = 0.05; ^d^ G C del/ G C del before vs G C del/ G C ins before: p = 0.015; ^e^ G C del/ G C del before vs G C ins/ G C ins before: p = 0.013; ^f^ G C del/ G C del after vs G C ins/ G C ins after: p = 0.049; ^g^ G C del/ G C ins before vs after: p = 0.044; ^h^ G C del/ G C ins before vs G G del/ G C del before: p = 0.048;  ^i^ G C ins/ G C ins before vs G G del/ G C del before: p = 0.047

Comparison of diplotypes in long protocol: ^a^ A C del / A C del before vs A C del/ G C del before: p = 0.05; ^b^ A C del/ A C del before vs G C ins/ G C ins before: 0.03

Comparison of diplotypes G C del/ G C del, G C del/ G C ins and G C ins/ G C ins (short vs long cycle) by Kruskal - Wallis test, p = 0.03

**Supplementary Table 12** (Continued)

| **SHORT PROTOCOL** | | | | | | | | | | | | | | | | | | |
| --- | --- | --- | --- | --- | --- | --- | --- | --- | --- | --- | --- | --- | --- | --- | --- | --- | --- | --- |
| **Diplotype** | **A G del/**  **A G del** | | **A T del/**  **A C del** | | **G C del/**  **G C del** | | **G C del/**  **G C ins** | | **G C ins/**  **G C ins** | | **G G del/**  **A C ins** | | **G G del/**  **G C del** | | **G T ins/**  **G C del** | | **G T ins/**  **G G del** | |
| **Before or after IVF-ET** | **before** | **after** | **before** | **after** | **before** | **after** | **before** | **after** | **before** | **after** | **before** | **after** | **before** | **after** | **before** | **after** | **before** | **after** |
| Number of patients | 1 | 1 | 1 | 0 | 8 | 6 | 13 | 10 | 15 | 13 | 18 | 14 | 6 | 6 | 5 | 4 | 1 | 1 |
| Minimum | 70.66 | 50.78 | 51.39 | - | 52.20 | 36.90 | 0.0 | 0.0 | 2.375 | 1.776 | 0.0 | 0.0 | 6.607 | 43.20 | 24.90 | 33.92 | 218.3 | 102.6 |
| 25% Percentile | 70.66 | 50.78 | 51.39 | - | 63.30 | 55.43 | 2.370 | 49.72 | 3.239 | 12.43 | 25.38 | 23.90 | 38.30 | 46.55 | 26.28 | 40.59 | 218.3 | 102.6 |
| Median | 70.66 | 50.78 | 51.39 | - | **124.0 ^d, e^** | **143.3^f^** | **28.86^g, h^** | 113.9 | **61.20^i^** | 56.14 | 63.30 | 54.63 | 301.8 | 152.4 | 61.49 | 445.9 | 218.3 | 102.6 |
| 75% Percentile | 70.66 | 50.78 | 51.39 | - | 253.0 | 684.8 | 88.19 | 350.3 | 78.60 | 92.21 | 135.5 | 154.9 | 797.5 | 340.1 | 563.4 | 1167 | 218.3 | 102.6 |
| Maximum | 70.66 | 50.78 | 51.39 | - | 1429 | 1828 | 658.8 | 968.9 | 108.5 | 249.9 | 1315 | 2122 | 1054 | 396.1 | 758.1 | 1278 | 218.3 | 102.6 |
| Mean | 70.66 | 50.78 | 51.39 | - | 295.4 | 419.5 | 88.50 | 232.7 | 48.43 | 64.58 | 159.0 | 237.3 | 404.2 | 185.6 | 248.2 | 551.0 | 218.3 | 102.6 |
| Std. Deviation | 0.0 | 0.0 | 0.0 | - | 465.0 | 696.7 | 177.2 | 303.6 | 37.83 | 65.95 | 307.7 | 555.2 | 406.9 | 152.7 | 319.3 | 609.8 | 0.0 | 0.0 |
| Std. Error | 0.0 | 0.0 | 0.0 | - | 164.4 | 284.4 | 49.15 | 96.00 | 9.767 | 18.29 | 72.52 | 148.4 | 166.1 | 62.34 | 142.8 | 304.9 | 0.0 | 0.0 |
| Lower 95% CI of mean | 0.0 | 0.0 | 0.0 | - | -93.32 | -311.6 | -18.58 | 15.51 | 27.48 | 24.73 | 5.996 | -83.30 | -22.76 | 25.31 | -148.3 | -419.3 | 0.0 | 0.0 |
| Upper 95% CI of mean | 0.0 | 0.0 | 0.0 | - | 684.1 | 1151 | 195.6 | 449.9 | 69.38 | 104.4 | 312.0 | 557.9 | 831.2 | 345.8 | 644.7 | 1521 | 0.0 | 0.0 |
| D'Agostino & Pearson omnibus normality test K^2^ | N too small | N too small | N too small | N too small | 20.70 | N too small | 31.40 | 11.70 | 4.903 | 15.39 | 38.66 | 35.17 | N too small | N too small | N too small | N too small | N too small | N too small |

**Supplementary Table 12** (Continued)

| **LONG PROTOCOL** | | | | | | | | | | | | | | | | |
| --- | --- | --- | --- | --- | --- | --- | --- | --- | --- | --- | --- | --- | --- | --- | --- | --- |
| **Diplotype** | **A C del/**  **A C del** | | **A C del/**  **A G del** | | **A C del/**  **G C del** | | **A C del/**  **G G del** | | **A C ins/**  **A C del** | | **A C ins/**  **A C ins** | | **A C ins/**  **G C del** | | **A G del/**  **A G del** | |
| **Before or after IVF-ET** | **before** | **after** | **before** | **after** | **before** | **after** | **before** | **after** | **before** | **after** | **before** | **after** | **before** | **after** | **before** | **after** |
| Number of patients | 7 | 5 | 2 | 2 | 7 | 5 | 2 | 1 | 1 | 1 | 6 | 6 | 13 | 9 | 2 | 0 |
| Minimum | 36.35 | 0.0 | 2.668 | 1.529 | 0.0 | 0.0 | 63.38 | 79.41 | 83.11 | 87.53 | 35.70 | 9.637 | 2.109 | 33.49 | 74.45 | - |
| 25% Percentile | 103.4 | 12.51 | 2.668 | 1.529 | 23.19 | 9.215 | 63.38 | 79.41 | 83.11 | 87.53 | 40.69 | 31.68 | 39.37 | 35.05 | 74.45 | - |
| Median | **139.3^a, b^** | 31.86 | 82.42 | 88.17 | 81.26 | 19.76 | 77.77 | 79.41 | 83.11 | 87.53 | 48.87 | 57.30 | 104.9 | 61.12 | 108.7 | - |
| 75% Percentile | 299.3 | 399.5 | 162.2 | 174.8 | 108.0 | 96.85 | 92.16 | 79.41 | 83.11 | 87.53 | 220.6 | 384.0 | 366.5 | 192.7 | 142.9 | - |
| Maximum | 391.5 | 551.0 | 162.2 | 174.8 | 148.0 | 138.3 | 92.16 | 79.41 | 83.11 | 87.53 | 565.4 | 1115 | 1357 | 531.1 | 142.9 | - |
| Mean | 188.9 | 171.2 | 82.42 | 88.17 | 69.91 | 46.38 | 77.77 | 79.41 | 83.11 | 87.53 | 141.1 | 236.5 | 252.3 | 134.8 | 108.7 | - |
| Std. Deviation | 122.8 | 234.7 | 112.8 | 122.5 | 51.01 | 55.16 | 20.35 | 0.0 | 0.0 | 0.0 | 209.4 | 432.8 | 368.9 | 166.0 | 48.40 | - |
| Std. Error | 46.42 | 104.9 | 79.75 | 86.64 | 19.28 | 24.67 | 14.39 | 0.0 | 0.0 | 0.0 | 85.48 | 176.7 | 102.3 | 55.34 | 34.22 | - |
| Lower 95% CI of mean | 75.35 | -120.2 | -930.9 | -1013 | 22.73 | -22.11 | -105.1 | 0.0 | 0.0 | 0.0 | -78.59 | -217.7 | 29.40 | 7.170 | -326.2 | - |
| Upper 95% CI of mean | 302.5 | 462.5 | 1096 | 1189 | 117.1 | 114.9 | 260.6 | 0.0 | 0.0 | 0.0 | 360.9 | 690.7 | 475.3 | 262.4 | 543.5 | - |
| D'Agostino & Pearson omnibus normality test K^2^ | N too small | N too small | N too small | N too small | N too small | N too small | N too small | N too small | N too small | N too small | N too small | N too small | 22.81 | 13.26 | N too small | N too small |

**Supplementary Table 12** (Continued)

| **LONG PROTOCOL** | | | | | | | | | | | | |
| --- | --- | --- | --- | --- | --- | --- | --- | --- | --- | --- | --- | --- |
| **Diplotype** | **G C del/**  **G C del** | | **G C ins/**  **G C ins** | | **G G del/**  **A C ins** | | **G T ins/**  **A C ins** | | **G T ins/**  **G C del** | | **G T ins/**  **G G del** | |
| **Before or after IVF-ET** | **before** | **after** | **before** | **after** | **before** | **after** | **before** | **after** | **before** | **after** | **before** | **after** |
| Number of patients | 1 | 1 | 5 | 5 | 10 | 8 | 1 | 1 | 1 | 0 | 1 | 1 |
| Minimum | 43.24 | 53.66 | 24.46 | 34.60 | 0.0 | 2.334 | 16.23 | 27.88 | 283.4 | - | 57.19 | 376.1 |
| 25% Percentile | 43.24 | 53.66 | 30.84 | 34.81 | 36.64 | 29.94 | 16.23 | 27.88 | 283.4 | - | 57.19 | 376.1 |
| Median | 43.24 | 53.66 | 45.94 | 79.30 | 99.57 | 68.42 | 16.23 | 27.88 | 283.4 | - | 57.19 | 376.1 |
| 75% Percentile | 43.24 | 53.66 | 58.68 | 105.5 | 186.2 | 177.0 | 16.23 | 27.88 | 283.4 | - | 57.19 | 376.1 |
| Maximum | 43.24 | 53.66 | 70.02 | 108.1 | 206.7 | 259.1 | 16.23 | 27.88 | 283.4 | - | 57.19 | 376.1 |
| Mean | 43.24 | 53.66 | 45.00 | 71.97 | 104.3 | 99.14 | 16.23 | 27.88 | 283.4 | - | 57.19 | 376.1 |
| Std. Deviation | 0.0 | 0.0 | 16.69 | 35.62 | 77.54 | 89.17 | 0.0 | 0.0 | 0.0 | - | 0.0 | 0.0 |
| Std. Error | 0.0 | 0.0 | 7.466 | 15.93 | 24.52 | 31.53 | 0.0 | 0.0 | 0.0 | - | 0.0 | 0.0 |
| Lower 95% CI of mean | 0.0 | 0.0 | 24.27 | 27.74 | 48.87 | 24.59 | 0.0 | 0.0 | 0.0 | - | 0.0 | 0.0 |
| Upper 95% CI of mean | 0.0 | 0.0 | 65.72 | 116.2 | 159.8 | 173.7 | 0.0 | 0.0 | 0.0 | - | 0.0 | 0.0 |
| D'Agostino & Pearson omnibus normality test K^2^ | N too small | N too small | N too small | N too small | 4.274 | 1.436 | N too small | N too small | N too small | N too small | N too small | N too small |
